# A Phase II Randomized, Double-Blind, Placebo-Controlled Study of the Efficacy, Safety, and Tolerability of Arbaclofen Administered for the Treatment of Social Function in Children and Adolescents With Autism Spectrum Disorders: Study Protocol for AIMS-2-TRIALS-CT1

**DOI:** 10.3389/fpsyt.2021.701729

**Published:** 2021-08-24

**Authors:** Mara Parellada, Antonia San José Cáceres, Melanie Palmer, Richard Delorme, Emily J. H. Jones, Jeremy R. Parr, Evdokia Anagnostou, Declan G. M. Murphy, Eva Loth, Paul P. Wang, Tony Charman, Andre Strydom, Celso Arango

**Affiliations:** ^1^Institute of Psychiatry and Mental Health, Hospital General Universitario Gregorio Marañón, Madrid, Spain; ^2^Instituto de Investigación Sanitaria Gregorio Marañón, Madrid, Spain; ^3^Centro Investigación Biomédica en Red Salud Mental (CIBERSAM), Madrid, Spain; ^4^School of Medicine, Universidad Complutense, Madrid, Spain; ^5^Department of Child and Adolescent Psychiatry, Institute of Psychiatry, Psychology and Neuroscience, King's College London, London, United Kingdom; ^6^Child and Adolescent Psychiatry Department, Robert Debré Hospital, APHP, Paris, France; ^7^Human Genetics and Cognitive Functions, Institut Pasteur, Paris, France; ^8^Centre of Brain and Cognitive Development, Birkbeck College, London, United Kingdom; ^9^Population Health Sciences Institute, Newcastle University, Newcastle upon Tyne, United Kingdom; ^10^Great North Children's Hospital, Newcastle upon Tyne Hospitals National Health Service (NHS) Foundation Trust, Newcastle upon Tyne, United Kingdom; ^11^Complex Neurodevelopmental Disorders Service, Cumbria, Northumberland Tyne and Wear National Health Service (NHS) Foundation Trust, Newcastle upon Tyne, United Kingdom; ^12^Department of Pediatrics, University of Toronto, Toronto, ON, Canada; ^13^Holland Bloorview Kids Rehabilitation Hospital, Toronto, ON, Canada; ^14^Department of Forensic and Neurodevelopmental Sciences, Institute of Psychiatry, Psychology and Neuroscience, King's College London, London, United Kingdom; ^15^Sackler Institute for Translational Neurodevelopment, Institute of Psychiatry, Psychology and Neuroscience, King's College London, London, United Kingdom; ^16^Clinical Research Associates LLC, New York, NY, United States; ^17^Department of Psychology, Institute of Psychiatry, Psychology and Neuroscience, King's College London, London, United Kingdom; ^18^South London and Maudsley National Health Service (NHS) Foundation Trust, London, United Kingdom

**Keywords:** autism, arbaclofen, social function, randomized controlled trial, children, adolescent

## Abstract

**Background:** Autism Spectrum Disorder (ASD or autism) is characterized by difficulties in social communication and interaction, which negatively impact on individuals and their families' quality of life. Currently no pharmacological interventions have been shown to be effective for improving social communication in autism. Previous trials have indicated the potential of arbaclofen for improving social function among autistic children and adolescents with fluent speech. The AIMS2TRIALS-Clinical Trial 1 (AIMS-CT1) will examine whether arbaclofen is superior to placebo in improving social function and other secondary outcomes over 16 weeks, along with safety and tolerability profiles.

**Methods:** AIMS-CT1 is an international, multi-site, double-blind, parallel group Phase II randomized clinical trial. It will include 130 males and females aged 5:0–17:11 years, with a diagnosis of ASD and fluent speech. Eligible participants will be randomized on a ratio of 1:1 for a 16-week treatment period. Medication will be titrated over 5 weeks. The primary outcome is the effect on social function from weeks 0 to 16 measured on the Socialization domain of the Vineland Adaptive Behavior Scales, 3rd edition^TM^. Secondary outcome measures include the CGI–S (Clinical Global Impression–Severity), CGI–I (Clinical Global Impression–Improvement), other areas of adaptive function, social communication and other autism symptoms, co-occurring behavior problems and health-related quality of life. Genetic and electrophysiological markers will be examined as potential stratifiers for treatment response. Exploratory novel digital technologies will also be used to measure change, examining simultaneously the validity of digital biomarkers in natural environments. The safety and tolerability of the drug will also be examined. Our protocol is very closely aligned with a parallel Canadian trial of 90 participants (ARBA Study, US NCT number: NCT03887676) to allow for secondary combined analyses. Outcomes will be compared using both an Intent-to-reat and Per Protocol approach.

**Discussion:** The outcomes of this trial, combined with the parallel Canadian trial, will contribute to the evidence base for medications used to help social difficulties among young autistic individuals; demonstrate the capabilities of the AIMS-2-TRIALS network of academic centers to deliver clinical trials; and support future drug development.

**Clinical Trial Registration:** EudraCT number: 2018-000942-21 and ClinicalTrials.gov registry number: NCT03682978. Currently under protocol v.7.2, dated 20.11.2020.

## Background

Autism Spectrum Disorder (ASD or autism) is a highly heritable disorder with prevalence rates in childhood between 1 and 2% (1, 2). Clinical presentation of autistic symptoms typically becomes apparent in early childhood and includes impairments in social interaction and communication, and the presence of sensory anomalies and repetitive and stereotyped behaviors [DSM-5; (3)]. Clinical presentation is highly heterogeneous, depending mainly on age, language, global cognitive levels and accompanying behavioral and/or emotional difficulties (4). A coherent understanding of the neurobiology of autism has not yet been achieved (5). However, recent findings suggest that autism results, in most cases, from atypical neurodevelopmental processes that have their onset *in utero*, and on-going physiopathological mechanisms at molecular, cellular, and circuit levels (6).

The heterogeneity of ASD is not only great at the level of the clinical phenotype but also at the level of the underlying neurobiological mechanisms that may lead to diverse phenotypes. Therefore, different molecules will likely improve deviant behaviors in different subgroups of patients. The study of pathogenic *de novo* mutations in large consortia has led to the identification of several different biological mechanisms as particularly relevant in the etiology of ASD. The study of differential neuropathological pathways that lead to distinct phenotypes and the possibility of finding biomarkers that may index differential pathways or disease trajectories within ASD is one of the most prominent topics in the study of the etiology of ASD and one included in the main objectives of the AIMS-2 TRIALS network (https://www.aims-2-trials.eu/). AIMS-2-TRIALS (Autism Innovative Medicine Studies-2-Trials) began in June 2018 and will run until May 2023. Its purpose is to accelerate medicine development, in particular, precision medicine, via a network of connected scientists and stakeholders across Europe and beyond. The research programme includes a range of work packages and sub-studies led by different academic, scientific and industry professionals to explore how autism develops, from before birth to adulthood, and how this varies in this population. The main aim of the consortium is to study biological markers and relate them to specific phenotypes, which could ultimately benefit from tailored treatments. The consortium will test, in specific subpopulations, new and repurposed medicines to help with social difficulties, repetitive behaviors and sensory processing. Currently, evidence-supported treatment options for core symptoms of autism do not include any pharmacological intervention. The clinical and biological heterogeneity of ASD may partially underlie the lack of positive results in clinical trials. Attention has turned recently to mechanistically targeted treatments. Among the most evidence-supported mechanisms is an excitatory-inhibitory unbalance that affects in, as yet unknown way, the proper coordination of the GABA and glutamate action at the appropriate developmental stages (7).

Glutamate is the most prevalent excitatory neurotransmitter, while gamma-aminobutyric acid (GABA) is the most prevalent inhibitory neurotransmitter in the human brain. Disruptions in GABA-ergic or glutamate signaling have been associated also with other neurodevelopmental disorders including autism and Fragile X Syndrome (FXS), as well as epilepsy (8, 9). A disruption in the excitatory/inhibitory (E-I) ratio is proposed to characterize the autistic brain (9, 10), but the direction of any such imbalance is less clear (11). Dysfunction in GABA signaling has been related to autism-like stereotypies (12). Arbaclofen has been tested in three moderately-sized studies: one in autism, and two in fragile X syndrome. New biomarker investigations have shown that arbaclofen modulates binocular rivalry, which was previously found to be abnormal in autism (13). In healthy controls, they found that arbaclofen increased perceptual suppression relative to placebo, consistent with the understanding of excitatory:inhibitory dynamics in the visual circuits for binocular rivalry. McAlonan et al. (14) very recently reported that arbaclofen dose-dependently rescues differences in visual contrast perception in adults with autism. These psychophysical effects were accompanied by changes in functional connectivity in multiple cortical circuits.

Taking all this evidence together it seems promising to explore medications that target E-I imbalance. Indeed, previous studies with GABA modulators have shown the potential for improving core autistic symptoms. For example, a recent study from the EU-AIMS consortium reported that differences in E-I balance can be 'shifted' using a GABA acting drug (riluzole), and that abnormalities in functional connectivity can be “normalized” by targeting E-I, including autistic adults (15).

One particularly fruitful target may be GABA type B receptors (GABAB). GABAB are crucial for maintaining the E-I balance and pervasive defects in GABAB receptor expression and activity have been associated with autism and are postulated to contribute to co-morbid seizure activity and cognitive impairment (8).

Arbaclofen (previously known as STX209) is a selective GABAB receptor agonist that augments GABA-ergic activity, inhibits presynaptic release of glutamate, inhibits postsynaptic transmission, and modulates intracellular signaling (16–18). Arbaclofen is the active enantiomer of racemic baclofen, an EMA and FDA approved GABAB agonist for spasticity. Baclofen has demonstrated efficacy in treating hyperactivity and audiogenic seizure phenotypes in the fragile X knockout mouse (19, 20). FXS mice models have been found to exhibit deficient GABA-mediated inhibitory neurotransmission particularly notable in the amygdala (21) brain region associated with affective behaviors involving emotional understanding and social interaction. Through elevation of GABA-ergic inhibitory activity, arbaclofen might alleviate autistic symptoms associated with social anxiety and emotional hyperarousal.

A previous trial has investigated whether Arbaclofen improves social difficulties in autism. Seaside Therapeutics initially conducted an open-label, flexible-dose, 8-week Phase II trial of arbaclofen in 32 autistic children and adolescents. Participants were treated with up to 10 mg thrice a day of arbaclofen and reported broad beneficial effects on autistic symptoms with no significant safety or tolerability concerns (22). This study was followed by a randomized, double-blind, placebo-controlled Phase II trial of arbaclofen in 150 autistic individuals between 5 and 21 years of age (23). Following the 12 weeks treatment, participants on arbaclofen and placebo showed no difference on the primary outcome measure [Social Withdrawal/Lethargy subscale of the Aberrant Behavior Checklist; (24)], but showed a nominally significant advantage on the Clinical Global Impression – Severity (CGI–S; 24, 25) for the arbaclofen group. *Post-hoc* exploration showed that drug-related improvements tended to be greater among the more verbally fluent individuals; and per-protocol analysis revealed a nominally significant improvement in of the Socialization domain of the Vineland Adaptive Behavior Scales, 2nd edition [VABS−2; (25)] and when scored by the same clinician both pre- and post-intervention, as per protocol. Safety results showed generally good tolerability, with somnolence and affect lability being more frequent in the active arm of the study. Given the results shown, and in view of the high heterogeneity of the condition that may be masking overall group results, there is rationale to investigate whether more specific, targeted and homogeneous groups (i.e., verbally fluent individuals) may benefit from the treatment with arbaclofen. Therefore, even though with the evidence available so far it is impossible to disentangle whether the positive results on verbally fluent children, adolescents and adults are due to the age and/or language level of the individuals in this group or whether it is the appropriateness of the assessment instruments used, we built on secondary outcome analyses from the Seaside study, and a homogeneous group of verbally fluent individuals was the target of the current clinical trial.

## Objectives

The primary objective of AIMS-CT1 is to examine the effect of arbaclofen vs. placebo on social function and behavior, as assessed through the Socialization Domain of the Vineland Adaptive Behavior Scales, 3rd edition [VABS−3; (26)]. We hypothesize that arbaclofen will be superior in improving social function impairments when compared to placebo. The key secondary objective is to examine the effect of arbaclofen vs. placebo on global functioning, as measured by CGI-I. Other secondary objectives are to examine the effects on other areas of adaptive function (communication and daily living skills), social communication behavior and other autistic symptoms, co-occurring behavior problems and health-related quality of life. The safety and tolerability of arbaclofen vs. placebo will also be examined. An exploratory objective is to examine whether electrophysiology and sensory discrimination is associated with treatment response to obtain pilot evidence for a predictive biomarker for future trials. An optional DNA sample (with an specific informed consent form) may help us to identify possible response genetic markers. Furthermore, this study adds the use of a novel, exploratory digital biomarker component that will collect data in naturalistic settings with minimal burden for the families. The use of digital biomarkers aims to gather more objective measures of social interaction and understanding than caregivers- or self- reports. They consist of active tasks (social games on a smartphone), passive monitoring of daily behaviors and surveys to participants and caregivers.

## Methods

### Trial Design

**Figure 1:**
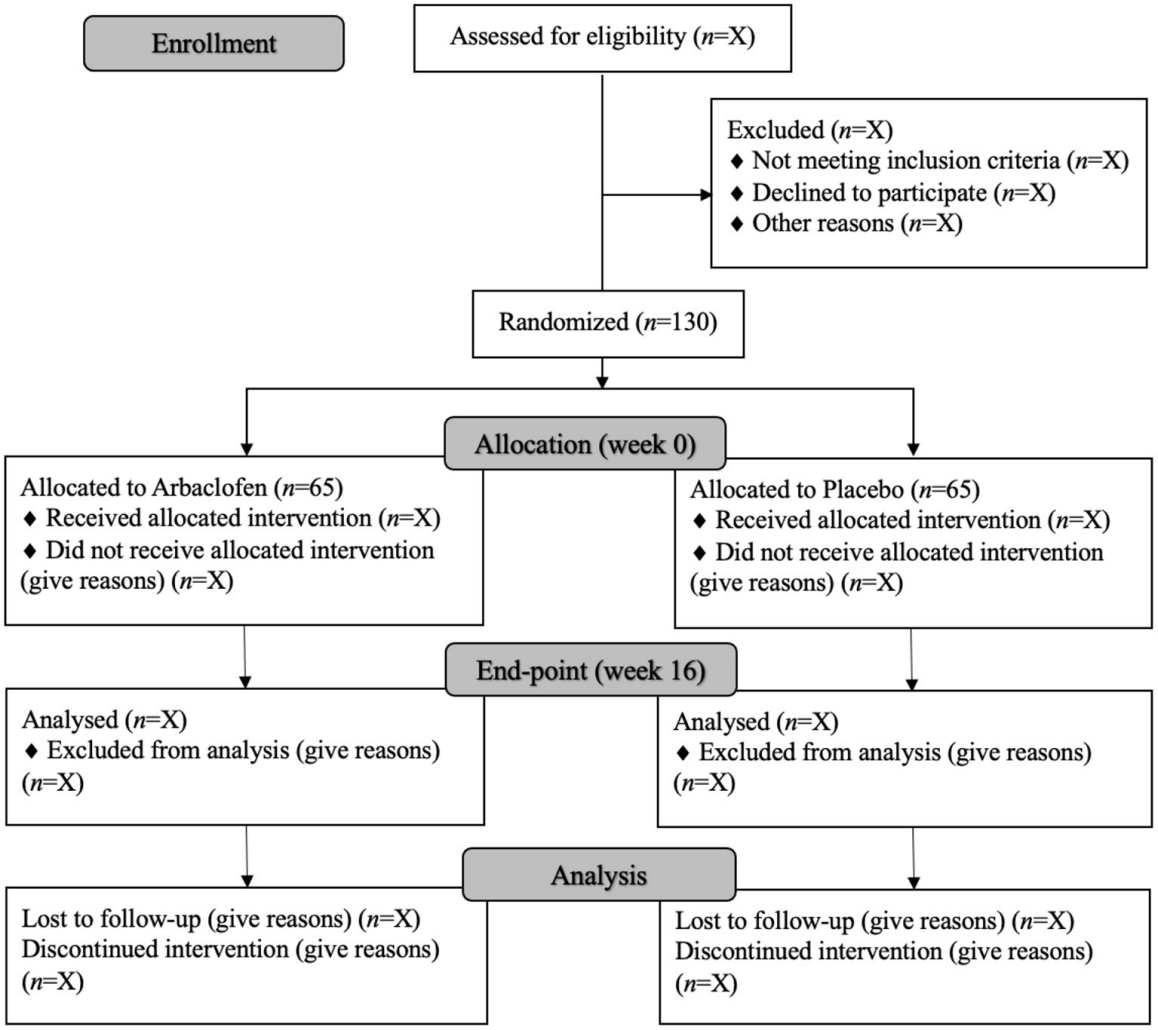
CONSORT diagram showing trial and participant flow through the trial (based on CONSORT guidelines).

The AIMS-CT1 is an international, multi-site, double-blind, parallel group randomized placebo-controlled Phase II trial. An Autism Representatives Group created in collaboration with Autistica's DISCOVER Research Network participated in several meetings along the trial and read and gave advice and opinion to the relevant final documents before these were submitted to the Regulatory Agencies. The study will examine the superiority of arbaclofen vs. placebo on the primary and secondary outcomes, along with safety and tolerability profiles over 16 weeks. Participants (*N* = 130) will be allocated to arbaclofen or placebo on a ratio of 1:1 and recruited across seven academic sites in Spain, UK and France. The trial design represents a refinement of the previous study of arbaclofen in ASD (23) and is summarized in Figure 1. In collaboration with the Province of Ontario Neurodevelopmental Disorders Network (POND) our protocol is very closely aligned with a parallel Canadian trial of 90 participants (ARBA Study: NCT03887676) for subsequent combination of data.

### Participants

#### Inclusion Criteria

- Male and female participants aged between 5:0 and 17:11 years at time of consent, with a diagnosis of ASD according to the DSM-5 (3) criteria and complex verbal language [defined as qualifying for an Autism Diagnostic Observation Schedule−2 (ADOS−2) Module 3 or 4 assessment (27), as determined by a clinically-certified ADOS-2 rater supervised by the local research-reliable ADOS-2 lead (see Requirements in Additional Material 5)].
- The parent/carer (hereafter parent; for brevity) can speak and understand the local language.
- The participant resides with the parent who will complete the primary outcome.
- Willingness to comply with the medication and research protocols.
- Pharmacological treatment affecting behavior stable for at least 6 weeks prior to screening, with no planned changes for the duration of the trial.
- Psychotherapeutic/psychosocial interventions affecting behavior stable for at least 3 months prior to screening, with no planned changes for the duration of the trial.
- Seizure history stable with anticonvulsant medication and seizure free for at least 6 months prior to screening. If anticonvulsant medication has not been stable for at least 3 months, the participant must be seizure free for at least 3 years prior to screening.
- Negative pregnancy test for female participants of childbearing potential.
- Female participants, and female partners of male participants, who are of childbearing potential and sexually active must agree to use highly effective forms of contraception.
- Participant able to take medication orally.

#### Exclusion Criteria

- Any medical condition that may interfere with the conduct of the trial, confound interpretation or endanger the participant's well-being. This includes but is not limited to impairment of renal function, evidence or history of malignancy, any significant hematological, endocrine, respiratory, hepatic, cardiovascular or gastrointestinal disease, or any clinically significant abnormalities on the electrocardiogram (ECG).
- Prohibited concomitant medications are those that include GABA agonists or modulators [e.g., vigabatrin, tiagapine, clobazam, regular benzodiazepine use (prn and hs use allowed), riluzole].
- Participants who have taken another investigational medicinal product in the 30 days prior to screening or have been involved in a previous trial of arbaclofen.
- A history of hypersensitivity to racemic baclofen.
- Rare hereditary problems of galactose intolerance, the lactase deficiency or glucose-galactose malabsorption.
- Porphyria.
- Active peptic ulceration.
- Engagement in illicit substance or alcohol abuse, according to DSM-5 criteria.
- Breastfeeding females.

Written informed consent will be signed by the participant's parent/carer/legal guardian. The participant will sign the informed consent form or assent form, following local laws and regulations.

### Interventions

Arbaclofen manufactured for this trial comes as round white orally disintegrating strawberry-flavored tablets with beveled edges, in strengths of 5, 10, 15, and 20 mg. It is stored at room temperature and will be packed in a box in blister cards of seven tablets. A flexible dose titration schedule will be utilized during the first 5 weeks of treatment (see Table 1). Dosing will be stratified by age. For participants aged 5–11 years at time of consent, the starting dose will be 5 mg once daily and increased to a maximum of 15 mg three times a day. For participants aged 12–17 years at consent, the starting dose will be 5 mg twice a day and increased to a maximum of 20 mg three times a day. Dosing titration is similar to Veenstra-VanderWeele et al. (23) with the addition of one higher dose to be tested in adolescents. If a participant does not tolerate a dose increase, under clinicians' advice, he or she should return to the previous dose level and remain at that level for the remainder of treatment. No change will be made to dosing after the 5-week titration period, unless for safety reasons. The treatment period of 16 weeks is 33% longer than in the study of Veenstra-VanderWeele et al. (23), in which the numerical difference between arbaclofen and placebo groups on the CGI-S had not plateaued at 12 weeks. At the end of treatment, the study medication will be tapered down over a period of up to 13 days.

**Table 1:** Dosing and tapering down regime for arbaclofen stratified by age.

**Timepoint**	**Age of participant**			
	**5–11 years**		**12–17 years**	
	**Strength**	**Frequency of administration**	**Strength**	**Frequency of administration**
Week 1 (Day 1, V1)	5 mg	Once daily	5 mg	Twice daily
Week 2 (Day 8)	5 mg	Twice daily	10 mg	Twice daily
Week 3 (Day 15, V2)	10 mg	Twice daily	10 mg	Three times a day
Week 4 (Day 22)	10 mg	Three times a day	15 mg	Three times a day
Weeks 5–16 (Day 29+, V3)	15 mg	Three times a day	20 mg	Three times a day
**Tapering down regime for those on maximum dose at week 16, V7**				
Days 113–116, (V7+3 days)	10 mg	Three times a day	15 mg	Three times a day
Day 117–119	10 mg	Twice daily	10 mg	Three times a day
Day 120–122	5 mg	Twice daily	10 mg	Twice daily
Day 123–125	5 mg	Once daily	5 mg	Twice daily
Day 126	None	–	None	–

*V, Visit*.

The comparator is a placebo tablet. The placebo tablets will have a similar shape, mass, color, smell and taste to the arbaclofen tablets and be provided in identical blister cards of seven tablets in an identical box. The placebo tablets will be administered at the same frequency as described above for arbaclofen and stratified by age. The packs will be released to a blinded study team member for dispensing.

### Randomization and Allocation

Randomization of participants will be performed 1:1 into arbaclofen vs. placebo by a randomization website (Interactive Web Response System—IWRS) developed by the Data Management Department of the Julius Center, University Medical Center Utrecht (UMCU). Allocation will be sent to an unblinded dedicated pharmacist at each individual site as specified in a separate Pharmacy Manual. A central unblinded monitor will double check dispensation regularly. The rest of the study members are blind to allocation. Randomization will be stratified by site and age group (5–11 years old; 12–17 years old). Unblinding will only happen if the information can help treat an adverse event and for safety reasons.

### Outcomes

Table 2 describes all outcomes, safety and sample characterization measures used in the trial. Following *post-hoc* findings in Veenstra-VanderWeele et al. (23), the primary outcome measure chosen is social function as measured by the Vineland Adaptive Behavior Scales, Third Edition (VABS-3)– socialization domain (26) administered by the same rater along the trial, preferably blinded to other outcome measures within a given patient.

**Table 2:** List of study measures.

**Name of measure**	**Measure details**
**Primary outcome**	
Socialization domain of the Vineland Adaptive Behavior Scales, 3rd edition (VABS−3) (26)	The Socialization Domain of the VABS−3 measures social function and behavior, covering interpersonal relationships, play and leisure and coping skills. The informant (parent/carer) is required to reside with the participant. The same informant and interviewer will be used at both timepoints, and any change will be reported as a protocol deviation, and wherever possible the interviewer will be blind to other study assessments. Interviewers administering the VABS−3 will be required to achieve 90% reliability with a gold standard rater in order to administer it for the trial. Regular reliability meetings will be scheduled for VABS−3 gold standard raters and interviewers across study sites to maintain reliability.
**Key secondary outcomes**	
Clinical Global Impression – Severity (CGI–S) scale (28, 29)	The CGI–S will assess the severity of impairment in global functioning, including but not limited to social engagement, internalizing and externalizing problems. It is rated on a 7-point scale ranging from 1 (normal, not at all impaired) to 7 (among the most extremely impaired) by a treating clinician. A CGI-S score of 3 (mildly impaired) will be the anchor applied to all participants meeting diagnostic criteria for ASD, with higher scores indicating significant co-occurring problems.
Clinical Global Impression – Improvement (CGI–I) scale (28, 29)	The Clinical Global Impression – Improvement (CGI–I) scale will measure improvement in global functioning during the previous week since treatment initiation. It will be rated by a treating clinician on a 7-point scale ranging from 1 (very much improved) to 7 (very much worse), with 4 representing no change. All available information will be used to inform clinical judgement. The CGI–I scores will also be used to assess safety, with participants who have CGI–I scores of 6 or more (much or very much worse) for two or more consecutive visits being discontinued from the trial.
**Other secondary outcomes**	
Communication and Daily Living Skills domain of the Vineland Adaptive Behavior Scales, 3rd edition (VABS−3) (26).	The Communication and Daily Living Skills domains of the VABS-3 (described in primary outcome above) will be administered to assess these areas of adaptive behavior.
Brief Observation of Social Communication Change (BOSCC) (30)	The BOSCC is a brief, 12-min observation of a semi-structured social interaction between the participant and an examiner. The interaction is video-recorded, and autism symptoms are coded using an algorithm.
Social Responsiveness Scale, 2nd edition (SRS−2) (31)	The SRS−2 is a 65-item measure identifying the presence and severity of autistic symptoms. Items are rated on a 4-point scale with higher scores reflecting more severe autism. It will be completed by parents/carers and teachers. The teacher completing this measure should spend at least 10 h per week in direct contact with the participant.
Autism Impact Measure (AIM) (32)	The AIM will be completed by the parent/carer to assess symptoms of autism. It consists of 41 items measuring both the frequency and impact of autism symptoms using a 5-point scale.
Aberrant Behavior Checklist - Community version (ABC-C) (24)	The ABC-C will also be completed by the parent/carer to measure irritability, lethargy/social withdrawal, stereotypic behavior, hyperactivity/non-compliance, and inappropriate speech displayed by the participant. Fifty-eight items are rated on a 4-point scale, with higher scores indicating more severe problem behaviors. The ABC-C will be completed by the parent/carer.
Child Behavior Checklist (CBCL) (33)	The CBCL will be used to measure emotional and behavioral problems. Items are rated on a 3-point scale with higher scores indicating more problems. Two different versions will be administered to parents of 5-year olds (1–5-year version) and 6–17-year olds (6–18-year version).
Pediatric Quality of Life Inventory (PedsQL) (34)	The PedsQL measures health related quality of life in children and adolescents. The Generic Core Scales, consisting of 23 items rated on a 5-point scale, will be used to measure physical and psychosocial health. Parents/carers will complete one of three different versions dependent on the participant's chronological age (5–7; 8–12; 13–18 years).
**Exploratory measures**	
Electrophysiology and sensory processing	Electrophysiology and sensory processing will be measured using electroencephalograms (EEG). Computer tasks chosen specifically for the trial will be completed whilst the EEG is being conducted to measure resting state, response to social and non-social stimuli, auditory processing and habituation response. The tasks take about 1 h to complete.
DNA acquisition	DNA samples (using 6 mls EDTA tubes) will be obtained from patient, and both parents when possible. This will help us to explore the genetics of those responders to the drug, vs. non-responders.
Digital Biomarkers	Digital biomarker (dBM) technology allows the remote measurement of the signs and symptoms of ASD, which can potentially reduce the burden of site visits and allow frequent/daily tracking in an ecologically valid environment. Patients will be asked to complete some tasks on a pre-set mobile phone and wear a smart wrist watch during the time of the study. Bluetooth beacons will be used to estimate the frequency of social interactions at home.
**Safety assessments**	
Medical checks	Vital signs (pulse, temperature and non-supine blood pressure) will be checked by a treating clinician. Physical examinations and electrocardiograms will be performed, and height and weight will be recorded with all outer wear and shoes removed. Safety blood tests checking complete blood count, liver enzymes, renal function and non-fasting glucose will be performed. Pregnancy testing will be performed for female participants of childbearing potential. Drug testing will also be performed on urine samples.
Safety Monitoring Uniform Report Form (SMURF) (35)	The SMURF will be administered by a treating clinician to record possible adverse events of psychotropic medication. Where appropriate, the participant and their parent/carer will be interviewed together and events since the last visit will be sought.
Epworth Sleepiness Scale for Children and Adolescents (ESS-CHAD) (36)	The ESS-CHAD will be administered by a treating clinician to measure daytime sleepiness and sedation in the past week. It consists of 8 items tapping into different situations which are rated on a 4-point scale with higher scores indicating greater chance of sleepiness.
Columbia-Suicide Severity Rating Scale (C-SSRS) (37)	Suicidality assessments will be completed by a treating clinician using the C-SSRS to measure the presence and intensity of suicidal ideation and behavior. The “baseline” version will be administered at week 0 and the “since last time” version thereafter. Two different versions will be administered depending on the chronological age of the participant.
**Screening/characterization assessments**	
Autism Diagnostic Observation Schedule−2 (ADOS−2) (27)	The ADOS−2 modules 3 or 4 will be administered to enable characterization of autism symptoms and severity. If a reliable ADOS−2 assessment has been administered in the 24 months prior to screening, with family consent, the scores from the previous assessments will be used an administration of these assessment will not be required.
Wechsler Scales	The appropriate Wechsler Scales according to age will be administered to enable characterization of the cognitive functioning of the sample. If a reliable standardized cognitive assessment has been administered in the 24 months prior to screening, with family consent, the scores from the previous assessments will be used an administration of these measures will not be required.
Social Communication Questionnaire-Lifetime version (SCQ) (38)	The SCQ is a 40-item yes-no measure of autism symptoms and severity and will be completed by the parent/carer. Scores of 15 or greater indicate a possible ASD.
Repetitive Behavior Scale-Revised (RBS-R) (39)	This questionnaire is part of the digital biomarkers optional sub-study and only completed if opted in. The RBS-R is an empirically-derived comprehensive survey of the entire spectrum of repetitive behaviors clinically observed and referred to in the DSM-IV (3) diagnostic description of Autistic Disorder. Parents or caregivers rate 43 behaviors on a scale of 0–3, where 0 indicates the behavior does not occur and 3 indicates the behavior does occur and is a severe problem.

### Sample Size

Based on Veenstra-VanderWeele et al. (23), for a 5.3 (standard deviation of 15) change from weeks 0 to 16 in scores on the VABS−3 Socialization domain for those treated with arbaclofen vs. placebo, 100 participants are needed in each arm, assuming 2-sided testing at a significant level of 5 and 80% power. Allowing for 10% attrition, the total sample required would be 220, 110 in each treatment arm. Therefore, the sample from this study will be combined with that of the Canadian ARBA trial to attempt to answer to the key objective. AIMS-CT1 will recruit 130 participants across the seven study sites (i.e., ~19 participants each) and ARBA-Brain Canada will recruit 90 participants.

### Recruitment

Recruitment of participants will be through referral via local autism diagnostic teams and pediatric and Child and Adolescent Mental Health Services at each study site. Consent databases/registers, support groups and advertisements on relevant websites may also be used. Potential participants can also self-refer. After initial contact by attending physician, and provided the participant/legal tutor give consent to be contacted, a study member will contact with the parent/carer and potential participant for pre-screening for eligibility, and, if appropriate, families will be invited to attend a screening visit to be assessed for eligibility and discuss consent. Principal investigator or delegated research team physicians will explain and subsequently obtain informed consent/assent. If patient/family agrees to participate, a schedule for the whole trial will be agreed with them, in order to accommodate families' needs and availability and make the completion of the trial more plausible. Privacy laws and regulations will be adhered to during all procedures related to this study. The collection and processing of participants' personal information will be limited to what is necessary to insure the study's scientific practicability. The local investigator or her/his co-workers will collect data and transfer it without recording the patient's name or date of birth coded with a patient identification number. A patient identification code list linking the individual patients to the identification numbers will be kept at the site; access is restricted to authorized study team members.

### Assessments

Table 3 shows the schedule for enrolment, treatment and the visits for participants, including all the assessments conducted at each visit. Participation in the trial will consist of a screening visit followed by eight visits for a period of 18 weeks. Table 3 shows an overview of the timeline and specific assessments conducted at each visit. To check the adherence, the left over pills get accounted for at all visits and crosschecked with the compliance calendar for medicine intake given to the families.

**Table 3:** Participant timeline showing schedule of enrolment, treatment and visits for participants.

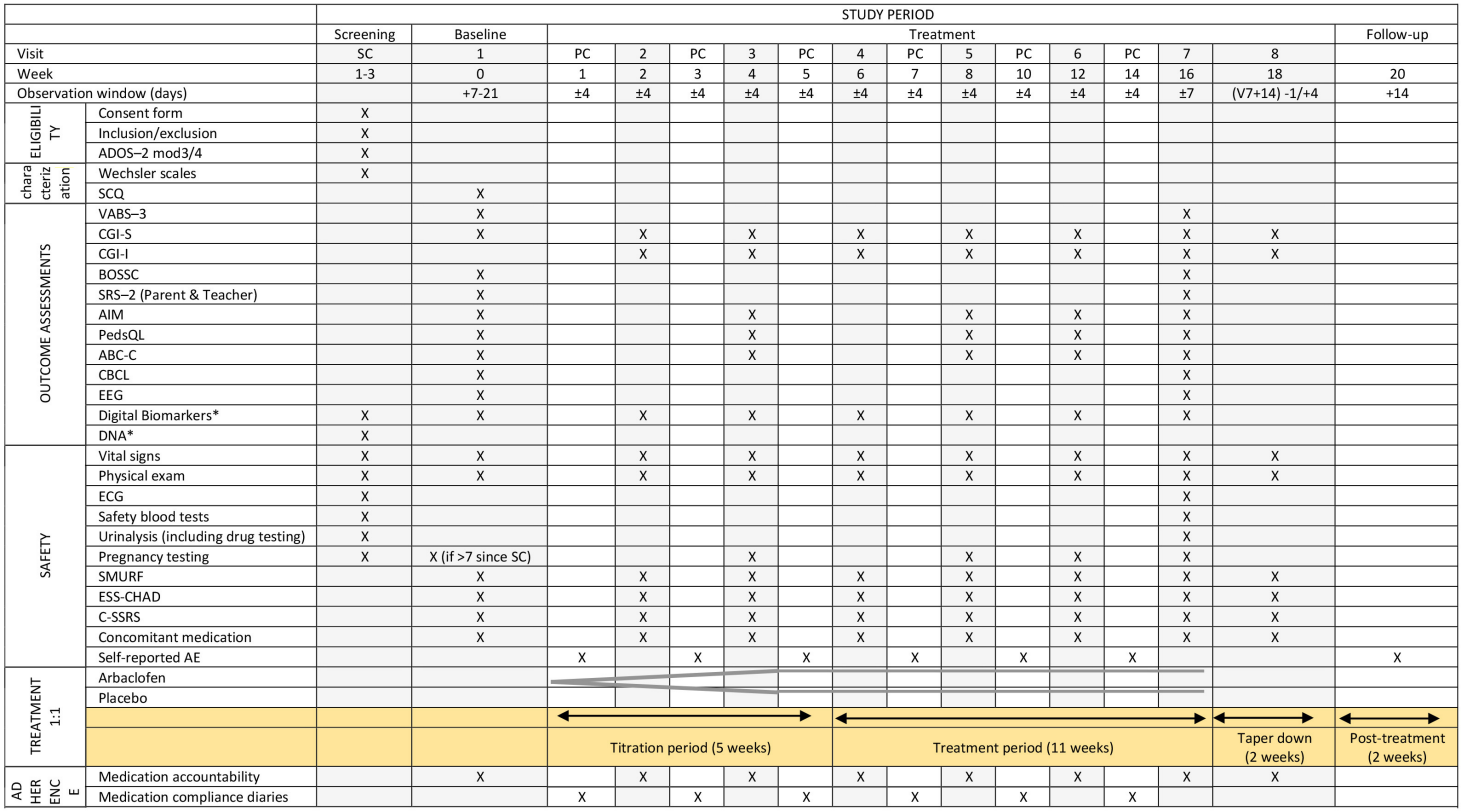

*Columns shaded in gray are the face-to-face visits. ABC-C, Aberrant Behavior Checklist-Community; ADOS−2, Autism Diagnostic Observation Schedule, 2nd edition; AIM, Autism Impact Measure; BOSCC, Brief Observation of Social Communication Change; CBCL, Child Behavior Checklist; CGI-S, Clinical Global Impression-Severity; CGI-I, Clinical Global Impression-Improvement; C-SSRS, Columbia-Suicide Severity Rating Scale; ECG, Electrocardiogram; EEG, Electroencephalogram; ESS-CHAD, Epworth Sleepiness Scale for Children and Adolescents; PC, Phone call; PedsQL, Pediatric Quality of Life Inventory SC, Screen; SCQ, Social Communication Questionnaire; SMURF, Safety Monitoring Uniform Report Form; SRS−2, Social Responsiveness Scale, 2nd edition; VABS−3, Vineland Adaptive Behavior Scales, 3rd edition*.

* *optional [the digital biomarkers include the completion of one small cognitive task every day, the completion by the parent of the RBS-R questionnaire (39) at visits 1 and 7, and the completion of satisfaction questionaries at visit 7; for DNA, samples will be also collected from both parents if applicable]*.

The trial includes a targeted electroencephalogram (EEG) battery designed to capture sensitive and predictive biomarkers of treatment efficacy at a level that is putatively closer to the underlying neural systems affected by arbaclofen. EEG measures the coordinated electrical activity of pyramidal cells in the outer cortical layers (40).

The task battery (see Additional Material 1 for full information) is designed to tap potential electrophysiological effects of arbaclofen on the (1) excitatory-inhibitory balance of cortical neural activity, and (2) brain specialization for social processing that may relate to the social functioning targeted in the trial, manifested by brain responses to social stimuli (41, 42).

An optional blood sample for DNA extracting is collected from participants and his/her parents that specifically consent to this. Samples are then sent for analyses to Institut Pasteur (Paris, France) where is and safely stored for 25 years after the explicit consent of the patient/legal/parents with a personal code with no connection with any personal information. Also optional, is the inclusion of digital biomarkers (see Additional Material 1), to obtain pilot evidence for treatment responsive biomarkers relevant to core and associated symptoms of autism.

All the procedures undertaken and all relevant clinical information is collected both in the electronic CRF and the electronic medical record.

### Statistical Analysis

Analysis will be performed both with an Intent to Treat (ITT) and as Per Protocol (PP) approach. The initial efficacy analysis will use the ITT sample, which includes all participants randomized who have at least one dose of medication and one post-baseline assessment. To be included in the PP sample, participants will fulfill ITT criteria and have attended at least 80% of the visits, have taken at least 70% of the prescribed medication and have a consistent pre- and post-intervention VABS−3 clinician rater.

Efficacy will be indicated by a difference between treatment arms at week 16, using Analysis of Covariance (ANCOVA; with follow-up score used as the dependent variable and treatment group as a factor) or logistic regression, and Chi-Square techniques, as appropriate. Age will be adjusted for and baseline scores will be used as covariates. Hypothesis testing will be performed at the 5% level of significance for 2-sided tests. Safety analyses will be conducted on all participants taking at least one dose of study medication by calculating the incidence of adverse events in each arm and summarizing laboratory and ECG assessments, physical examinations and vital signs.

A secondary analysis will be conducted by combining data from AIMS-CT1 and the ARBA Study to ensure statistical power for the assessment of efficacy of the primary outcome variable. Additional analyses will consider variables or sub-variables that will be different from or derivative of the primary and secondary hypotheses. Another level of analysis will look at data of individual sites. Power calculation was made for the primary outcome (VABS-3 social function domain), which justifies the a priori planned combination of the data of this study with that of ARBA Study in Canada. Other results will be considered secondary results and therefore more exploratory and hypotheses-generating.

While no interim analysis is planned, if necessary, the Data Monitoring Committee (DMC) will consider it and inform the Sponsor whether premature termination criteria are met.

### Adverse Event Reporting

A medical history and physical examination will be conducted at screening to assist with interpretation of any adverse events (AEs), whether serious (SAEs) or non-serious, that occur during participation in the trial. The standard definition of AEs and SAEs will apply. In addition, pregnancy (in participant, or female partners of male participants), overdose (either accidental or intentional), cancer, potential drug-induced liver injury and suspected transmission of an infectious agent will be recorded as SAEs and follow these reporting regulations. In all cases of pregnancy, the treatment will be permanently discontinued in an appropriate manner and follow-up information on the course of pregnancy should be recorded if permission is given by the participant.

AEs and SAEs may be reported by the participant and/or their parent or found through medical examination or laboratory testing during phone call monitoring or face-to-face visits. The nature of each event, onset, duration and severity will be established and recorded, regardless of whether the event is related to the study treatment or not. AEs will be recorded from the initiation of study treatment until the final visit. All SAEs that occur between screening and up until 30 days of the last dose of the study treatment will be recorded. All AEs and SAEs will be followed-up proactively at subsequent visits/phone calls until either resolution, the condition stabilizes, the event is otherwise explained, or the participant is lost to follow-up. Follow-up is also required for AEs that cause interruption or discontinuation of the study treatment.

Usual convention will be used for reporting all AEs and SAEs following local laws and regulations. All SAEs will be reported to the sponsor and sponsor delegate within 24 h of the study site becoming aware of the event. Any Suspected, Unexpected Serious Adverse Reactions (SUSARs) will be reported to the appropriate regulatory authorities following local and global guidelines and requirements.

### Context

#### AIMS-2-TRIALS Network

AIMS-CT1 is one of the many studies taking place within the AIMS-2-TRIALS network (www.aims-2-trials.eu), which aims to apply a precision medicine approach to ASD and improve patient outcomes by tailoring treatments to a patient's biological profile. Building on the achievements of other IMI (Innovative Medicines Initiative) initiatives, Horizon 2020 networks, and SMEs (Small Medium Enterprise), the specific objectives are to validate and qualify stratification biomarkers from infancy to adulthood; develop objective outcome measures that can be used in trials; create a European-wide clinical trials network that reliably carries out studies able to support filings to the European Medicines Agency/ Food and Drug Agency (EMA/FDA); to carry out better targeted clinical trials linked to other international efforts—including quick wins or “fast fails” of ineffective agents—and to translate molecular mechanisms and drug effects between preclinical models and particular subtypes of ASD.

#### COVID Pandemic Adjustments

In March 2020, the WHO declared a COVID-19 global pandemic that would eventually affect health systems all over the globe. The European Medicines Agency (EMA) and the National Regulation Authorities, working with all relevant stakeholders, acknowledged the impact of the pandemic on the conduct of clinical trials and have been issuing additional guidelines for their management. The Sponsor and Principal Investigators of the present clinical trial have taken into account all relevant guidelines and local legislations and have made and reported to the authorities for their approval the appropriate adjustments to the design and conduct of the study. All these adjustments have been included in the updated versions of the Protocol. The most significant ones are: (i) recruitment of new participants was halted during the period that National global lockdowns were in place, (ii) some physical visits were converted into remote visits at the beginning of the pandemic; by phone and/or video-conferencing depending on the assessment instrument after authors/supervisors of the main evaluation instruments were consulted; in this case, vital signs were recorded at local pharmacies; primary outcome measure (social function by the VABS-3 social domain score) was conducted via videoconference at the time of lockdown or whenever it was more risky for the families to attend the hospital, by the same rater and with the same informant; the same format (video or in-site) was maintained for visit 1 and visit 7 for the same patient (iii) transferring of participants specific assessments (e.g., EEG) to investigational facilities away from risk zones or, if possible, to the participants' homes, (iv) transfer of sample extraction, medication provision (ensuring maintenance of temperature control) and other medical/nursing procedures to authorized local facilities and/or participants' homes if possible, (v) on-site monitoring transferred to hired local monitors (vi) local instructions followed at all times for screening for COVID-19 symptoms and enhanced social distancing and protection and cleaning regimes are in place as per site local instructions. For all decisions, a benefit-risk balance of the integrity of the trial and well-being of the participants was taken into consideration with safety always being the prevailing concern.

## Discussion

The AIMS-CT1 is a phase II double-blind, parallel group, randomized placebo-controlled trial designed to investigate the efficacy, safety and tolerability of arbaclofen for social function over 16 weeks in 5 to 17-years-old autistic males and females with fluent speech. It will also examine the superiority of arbaclofen vs. placebo on global functioning, other areas of adaptive functioning, social communication behavior, autistic symptoms, co-occurring behavioral problems and health-related quality of life. The outcomes of this trial, combined with the parallel Canadian ARBA Study, will contribute to the evidence base for medications used to help reduce social difficulties among young autistic individuals. Positive findings have the potential to improve the quality of life for young autistic individuals and those involved in their care. Improvements in social function in the short-term may lead to increased participation in social activities and better outcomes for young people with autism. If arbaclofen is found to improve social function, further trials are warranted, including for other medications targeting excitatory/inhibitory imbalances in autism.

Testing pharmacological treatments for autistic symptoms has been challenging because of the vast variation in phenotypic presentation and limited understanding of underlying causes. A key limitation of trials for autism is the generalizability of any effects of the tested drug to individuals across the whole spectrum, and in the case of this particular trial, specifically to those autistic individuals who have more significant cognitive impairment or less fluent verbal ability. Additional research is required to investigate this, given the additional impacts and costs co-occurring intellectual disability has on the individual, their family and society (43).

Design of the current trial was informed in two important ways by lessons learnt from the Veenstra-VanderWeele et al. (23). First, the primary outcome is the Socialization domain of the VABS−3 (26). Following results in Veenstra-VanderWeele et al. all efforts will be made to maintain the same interviewer and informant at both time points and any change in interviewer or informant will be reported as a protocol violation. When possible, the VABS−3 rater will be blind to other study assessments, otherwise this will be considered a protocol deviation. All interviews will be recorded for external monitoring, with a central gold standard rater per site that will review at least 25% of the recordings and provide feedback to raters. Specific training was provided within the trial team across sites to address the particular challenges of conducting an international study conducted in multiple languages, and the trial team consulted with a co-author (Dr. Celine Saulnier) of the instrument on training on the VABS−3.

Secondly, we employed an inclusion criterion for the participant to be verbally fluent (instead of using an IQ criterion). Previous pharmacological trials in autism have either enrolled participants across a wide range of functional skills, or restricted enrolment to individuals with IQ scores above a certain threshold [e.g., IQ ≥ 70; (44)]. The rationale for restrictive enrolment has been that drug effects might only be evident in the higher IQ subgroup. In fact, however, there are no mechanistic biological hypotheses to support such supposition for any drug or drug mechanism. Rather, any apparent disadvantage in response seen in individuals with weaker functional skills is likely related to limitations in outcome assessment for these individuals. Standardized cognitive and behavioral outcome measures often show limited resolution in lower ranges of development, as they are not intended or designed as measures of change in significantly impaired cohorts. *Post-hoc* analyses (unpublished) of the Seaside Therapeutics trial (23) of Arbaclofen showed numerically larger benefit in participants with stronger functional skills, regardless of whether those skills were defined by IQ, verbal fluency (ADOS module 3 or 4, vs. module 1 or 2), or Vineland Communication domain age-equivalent scores. AIMS-CT1 will employ the criterion for verbal fluency based on ADOS−2 module, which aligns with clinically meaningful distinctions in communicative function, rather than employing an arbitrary numerical threshold from some language assessment measure. If treatment results in improved social function, individuals who are verbally fluent will be able to manifest their improvement in communicative behaviors that are evident to their social partners and to observers, including parents, and can be quantified precisely on available psychoeducational measures. Based on our own meta-analyses (45), we included some of the variables (e.g., flexible dosing, reduced number of recruiting sites, threshold of baseline symptoms) to reduce placebo effect.

AIMS-CT1 was initiated prior to the COVID-19 pandemic. The pandemic led to a suspension of recruitment in all sites. In two sites that had commenced enrollment and randomized 14 participants, and in alignment with national and international official and expert-driven guidelines, procedures were adapted to limit face-to-face visits. It is unknown how the COVID-19 pandemic and lockdown policies that change dramatically the routines and interventions received by the participants in the trial and change the way patients are assessed during the trial will impact on the nature of data collected for these individuals along with future participants and we recognize there may be potential effects that will need to be monitored. However, randomization should mean that these issues will affect each arm equally. All protocol deviations or violations that occurred due to the pandemic obligations have been recorded in detail for report to authorities and to inform the monitoring board of the trial as has been recommended (46, 47). The experience of running a clinical trial through the pandemic has shown the importance of identifying the most important aspects within the trial in which strict procedures need to be followed and other procedures and/or measurements in which flexible acquisition or assessment procedures and their means can be anticipated per protocol. For this, experienced and reliable researchers, as those available in large academic centers, facilitate adaptation to unexpected circumstances.

## Ethics Statement

Ethical approval for the trial has been obtained from: France: Comité de Protection des Personnes (CPP) “Sud-Méditerranée IV” (Ref CPP: 19 09 04), L'Agence Nationale de Sécurité du Médicament et des produits de santé (ANSM, French Medicines Agency); Spain: CEIm Hospital General Universitario Gregorio Marañón, Madrid (Ref: #Cod. CNH: 280246# [Madrid]); Agencia Española del Medicamento y Productos Sanitarios (AEMPS); UK: East Midlands – Leicester Central Research Ethics Committee (Ref: 18/EM/0335) and UK Medicines and Healthcare products Regulatory Agency (MHRA). Written, informed consent will be given by the participant's parent/carer/legal guardian and participant, if applicable, in line with local laws and regulations. Assent from the participant will be obtained in all cases. An independent Data Monitoring Committee (DMC) comprising of expert clinicians and a statistician will provide oversight of the trial conduct and monitor safety, efficacy and dose escalations. DMC members: Professor Alessandro Zuddas, Università di Cagliari, Sardinia, Italy; Professor Benedetto Vitiello, Università degli Studi di Torino, Turin, Italy and John Hopkins University, Baltimore, MD, United States; Dr. Michael McIsaac, University of Prince Edward Island, Prince Edward Island, Canada (Statistician).

## Author's Note

Further information with regards to Trial status, Confidentiality and Dissemination can be found in online Additional Material 2, complete SPIRIT ckecklist can be found in Additional Material 3 and a model of Consent Form in Additional Material 4.

## Author Contributions

CA, MPar, PW, TC, DM, EJ, and EL: conception of the study, protocol writing, and obtained funding. MPar, ASJ, RD, JP, EJ, LM, EA, DM, EL, PW, TC, AS, CC, and TB: contribution to the development and design of the study. MPar, MPal, ASJ, PW, TC, AS, MM, FM, VP, PSh, and PSi: helped to draft the manuscript. All authors read and approved the final manuscript and involved in reviewing and revising the manuscript for content.

## AIMS-2-TRIALS-CT1 Group

Stephanie Antoun, Child and Adolescent Psychiatry Department, Robert Debré Hospital, APHP, Paris, France; Cristina Baeza, Instituto de Investigación Sanitaria Gregorio Marañón, Madrid, Spain; Ana Blazquez-Hinojosa, Centro Investigación Biomédica en Red Salud Mental (CIBERSAM), Spain; Department of Child and Adolescent Psychiatry and Psychology Hospital Clinic, Barcelona, Spain; Thomas Bourgeron, Human Genetics and Cognitive Functions, Institut Pasteur, Paris, France; Université Paris-Diderot, Paris, France; Mónica Burdeus, Instituto de Investigación Sanitaria Gregorio Marañón, Madrid, Spain; Christopher Chatham, F. Hoffmann-La Roche, Innovation Center Basel, Basel, Switzerland; Rosa Calvo-Escalona, Centro Investigación Biomédica en Red Salud Mental (CIBERSAM), Spain; Department of Child and Adolescent Psychiatry and Psychology Hospital Clinic, Barcelona, Spain; University of Barcelona, Barcelona, Spain; Josefina Castro-Fornieles, Centro Investigación Biomédica en Red Salud Mental (CIBERSAM), Spain; Department of Child and Adolescent Psychiatry and Psychology Hospital Clinic, Barcelona, Spain; University of Barcelona, Barcelona, Spain; Ariane Cartigny, Child and Adolescent Psychiatry Department, Robert Debré Hospital, APHP, Paris, France; Université Paris-Diderot, Paris, France Teresa Del Bianco, Centre for Brain and Cognitive Development, Birkbeck College, London, United Kingdom María Carnicer, Institute of Psychiatry and Mental Health, Hospital General Universitario Gregorio Marañón, Madrid, Spain; Suhas Hydros, F. Hoffmann-La Roche, Innovation Center Basel, Basel, Switzerland; Elianne Huijsman, Department of Psychiatry, University Medical Center Utrecht Brain Center, Utrecht University, Utrecht, Netherlands; Florentia Kaguelidou, Clinical Investigation Center, CIC1426, Robert Debré Hospital, APHP, INSERM, Paris, France; Anya Kaushik, South London and Maudsley National Health Service (NHS) Foundation Trust, London, United Kingdom; Andrea Koch, Child and Adolescent Psychiatry Department, Robert Debré Hospital, APHP, Paris, France; Université Paris-Diderot, Paris, France; Luisa Lázaro, Centro Investigación Biomédica en Red Salud Mental (CIBERSAM), Spain; Department of Child and Adolescent Psychiatry and Psychology Hospital Clinic, Barcelona, Spain; University of Barcelona, Barcelona, Spain; Aline Lefevre, Child and Adolescent Psychiatry Department, Robert Debré Hospital, APHP, Paris, France; Université Paris-Diderot, Paris, France; Michael Lindemann, Roche Pharma Research and Early Development, Roche Innovation Center Basel, Hoffmann-La Roche, Basel, Switzerland; Luke Mason, Centre for Brain and Cognitive Development, Birkbeck College, London, United Kingdom; Maria Megalogeni, Department of Forensic and Neurodevelopmental Sciences, Institute of Psychiatry, Psychology and Neuroscience, King's College London, London, United Kingdom; Farah Mgaieth, Department of Forensic and Neurodevelopmental Sciences, Institute of Psychiatry, Psychology and Neuroscience, King's College London, London, United Kingdom; Ana Moscoso, Child and Adolescent Psychiatry Department, Robert Debré Hospital, APHP, Paris, France; Université Paris-Diderot, Paris, France; David Nobbs, Roche Pharma Research and Early Development, Roche Innovation Center Basel, Hoffmann-La Roche, Basel, Switzerland; Valeria Parlatini, Department of Forensic and Neurodevelopmental Sciences, Institute of Psychiatry, Psychology and Neuroscience, King's College London, London, United Kingdom; Mallika Punukollu, Institute of Health and Wellbeing, University of Glasgow, Glasgow, United Kingdom; Pretesh Shah, South London and Maudsley National Health Service (NHS) Foundation Trust, London, United Kingdom; Pushpika Singappuli, South London and Maudsley National Health Service (NHS) Foundation Trust, London, United Kingdom; NIHR Maudsley Biomedical Research Center (BRC), United Kingdom; Lourdes Sipos, Institute of Psychiatry and Mental Health, Hospital General Universitario Gregorio Marañón, Madrid, Spain; Andrew Stanfield, University of Edinburgh, Old College, South Bridge, Edinburgh, United Kingdom; Margot Slot, Department of Psychiatry, University Medical Center Utrecht Brain Center, Utrecht University, Utrecht, Netherlands; Ángela Ulloa, Instituto de Investigación Sanitaria Gregorio Marañón, Madrid, Spain; Elena Urbiola, Institute of Psychiatry and Mental Health, Hospital General Universitario Gregorio Marañón, Madrid, Spain; Instituto de Investigación Sanitaria Gregorio Marañón, Madrid, Spain; Centro Investigación Biomédica en Red Salud Mental (CIBERSAM), Spain; Karen Walton-Bowen, Clinical Research Associates LLC, New York, NY, United States; Inge Winter-van Rossum, Department of Psychiatry, University Medical Center Utrecht Brain Center, Utrecht University, Utrecht, Netherlands.

## Conflict of Interest

MPar is consultant or has received honoraria or grants from for Exeltis and Servier. ASJ has served in an advisory or consultancy role for F. HoffmannLa Roche Ltd. and she is involved in clinical trials conducted by Servier. CC is a full-time employee of F. HoffmannLa Roche. PW was previously employed by Seaside Therapeutics and has served as an unpaid consultant for Roche. TC has received consultancy fees from F. Hoffmann-La Roche Ltd and Servier, and royalties from Sage Publications and Guilford Publications. DM has served on advisory boards for Roche and Servier. He has also received research funding from J+J. AS has consulted for AC Immune and Aelis Farma, and he is an advisory board member of ProMIS Neurosciences. CA has been a consultant to or has received honoraria or grants from Acadia, Angelini, Gedeon Richter, Janssen Cilag, Lundbeck, Minerva, Otsuka, Roche, Sage, Servier, Shire, Schering Plough, Sumitomo Dainippon Pharma, Sunovion, and Takeda. EA has received consultation fees from Roche and Quadrant, research funding from Roche, in-kind supports from AMO pharma, editorial Honoria from Wiley and book royalties from APPI and Springer, she holds a patent for the device, “Tully” (formerly Anxiety Meter) and she has received royalties from APPI and Springer. DN is an employee of F. Hoffmann-LaRoche Ltd. ML is a consultant for F Hoffmann–La Roche Ltd. (Basel Switzerland) on behalf of Inovigate (Basel Switzerland). KB was previously employed by Seaside Therapeutics. The remaining authors declare that the research was conducted in the absence of any commercial or financial relationships that could be construed as a potential conflict of interest.

## Publisher's Note

All claims expressed in this article are solely those of the authors and do not necessarily represent those of their affiliated organizations, or those of the publisher, the editors and the reviewers. Any product that may be evaluated in this article, or claim that may be made by its manufacturer, is not guaranteed or endorsed by the publisher.

## Abbreviations

ABC-C: Aberrant Behavior Checklist-Community version
ADHD: Attention-Deficit/Hyperactivity Disorder
ADOS−2: Autism Diagnostic Observation Schedule, 2^nd^ Edition
AE: Adverse Event
AIM: Autism Impact Measure
ANCOVA: Analysis of Covariance
ASD: Autism Spectrum Disorder
BOSCC: Brief Observation of Social Communication Change
CBCL: Child Behavior Checklist
CGI–I: Clinical Global Impression – Improvement
CGI–S: Clinical Global Impression – Severity
C-SSRS: Columbia-Suicide Severity Rating Scale
CONSORT: Consolidated Standards of Reporting Trials
DMC: Data Monitoring Committee
DSM-5: Diagnostic and Statistical Manual of Mental Disorders, 5th Edition
ECG: Electrocardiogram
EEG: Electroencephalogram
E-I: Excitatory/Inhibitory
ESS-CHAD: Epworth Sleepiness Scale for Children and Adolescents
FXS: Fragile X Syndrome
GABA: Gamma-Aminobutyric Acid
GABAB: GABA type B
IRB/IEC: Institutional Review Board/Independent Ethics Committee
ITT: Intent-to-Treat
IWRS: Interactive Web Response System
PedsQL: Pediatric Quality of Life Inventory
POND: Province of Ontario Neurodevelopmental Disorders Network
PP: Per Protocol
PRN: Pro Re Nata
SAE: Serious Adverse Event
SAP: Statistical Analysis Plan
SCQ: Social Communication Questionnaire
SMURF: Safety Monitoring Uniform Report Form
SRS−2: Social Responsiveness Scale, 2^nd^ edition
SUSAR: Suspected, Unexpected Serious Adverse Reactions
UMCU: University Medical Center Utrecht
VABS−2: Vineland Adaptive Behavior Scales, 2nd Edition
VABS−3: Vineland Adaptive Behavior Scales, 3rd Edition.
