# Supplementary material for: A Phase II Randomized, Double-Blind, Placebo-Controlled Study of the Efficacy, Safety, and Tolerability of Arbaclofen Administered for the Treatment of Social Function in Children and Adolescents With Autism Spectrum Disorders: Study Protocol for AIMS-2-TRIALS-CT1
Source: Front Psychiatry. 2021 Aug 24;12:701729. doi: 10.3389/fpsyt.2021.701729 (PMC8421761; doi:10.3389/fpsyt.2021.701729)
Supplement: Supplementary file 1 [file Data_Sheet_1.docx]

# Additional material 1

# EEG battery

The trial includes a targeted electroencephalogram (EEG) battery designed to capture sensitive and predictive biomarkers of treatment efficacy at a level that is putatively closer to the underlying neural systems affected by arbaclofen. EEG measures the coordinated electrical activity of pyramidal cells in the outer cortical layers Kirschstein & Köhling, 2009. We used a 20‑electrode gel‑based mobile EEG (Neuroelectrics, Barcelona, Spain [www.neuroelectrics.com](http://www.neuroelectrics.com)), referenced to one earlobe\mastoid, and with a dynamic range of 0.05 µV and millisecond‑level resolution. We have standardised stimulus presentation and data acquisition hardware and software across contributing centres to avoid site effects, and will use an automated processing and analytic pipeline (TaskEngine) to ensure results are robust and reproducible. The task battery (Table App1) is designed to tap potential electrophysiological effects of arbaclofen on the 1) excitatory‑inhibitory balance of cortical neural activity (e.g., gamma‑band synchronization Fries, 2009; spectral composition, e.g., 1/f balance measured during ‘rest’, Ouyang et al., 2020), 2) brain specialisation for social processing that may relate to the social functioning targeted in the trial, manifested by brain responses to social stimuli (e.g. N170;Eimer, 2011).

1. Kirschstein, T., & Köhling, R. (2009). What is the source of the EEG?. Clinical EEG and neuroscience, 40(3), 146-149.
2. Neuroelectrics, Barcelona, Spain.
3. Fries, P. (2009). Neuronal gamma-band synchronization as a fundamental process in cortical computation. Annual review of neuroscience, 32, 209-224.
4. Ouyang, G., Hildebrandt, A., Schmitz, F., & Herrmann, C. S. (2020). Decomposing alpha and 1/f brain activities reveals their differential associations with cognitive processing speed. NeuroImage, 205, 116304.
5. Eimer (2011): The face-sensitive N170 component of the event-related brain potential. The Oxford handbook of face perception, 28, 329-44.

*Additional Table 1: Brief description of the tasks included in the EEG battery*

| Task | Brief Description | Dependent Variables | Domain and Effect | Reference |
| --- | --- | --- | --- | --- |
| Auditory Steady State | Presents trains of auditory tones, repeating clicks of white noise at  10Hz and 40Hz | EEG power at  each frequency | Sensory processing  Connectivity and frequency: Gamma Band Synchronisation | Rojas, Teale, Maharajh et al., (2011): Transient and steady‑state auditory gamma‑band responses in first‑degree relatives of people with autism spectrum disorder. Mol Aut 2, 11  Seymour, Rippon, Gooding‑Williams et al., (2020): Reduced auditory steady state responses in autism spectrum disorder. Mol Aut 11, 56 |
| Visual Steady State | Colourful icons flickering at 6 Hz, 10Hz, 15Hz | EEG power at  each frequency | Sensory processing  Connectivity and frequency: Gamma Band Synchronisation | Vialatte, Maurice, Dauwelsc et al., (2010): Steady‑state visually evoked potentials: Focus on essential paradigms and future perspectives, Prog Neurobiol 90(4), 418‑438  Snijders, Milivojevic, & Kemner (2013). Atypical excitation–inhibition balance in autism captured by the gamma response to contextual modulation. NeuroImage: Clin, 3, 65‑72 |
| Social and Non‑Social Videos (resting state) | Presents 1) a social  video – women singing – , and 2) a non‑social video – toys in motion – repeated twice | EEG power and  connectivity in all  frequency bands | Social processing  Connectivity and frequency: Alpha band Synchronization | Jones, Venema, Lowy et al., (2015). Developmental changes in infant brain activity during naturalistic social experiences. Dev Psychobiol 57(7), 842‑853  Jones, Goodwin, Orekhova et al., (2020): Infant EEG theta modulation predicts childhood intelligence. Sci Rep 10: 11232 |
| Resting State | Presents 1) abstract video 2) a fixation cross | EEG power and  connectivity in all  frequency bands | Connectivity and frequency: Spontaneous Synchronization | Murias, Webb, Greenson et al., (2007): Resting state cortical connectivity reflected in EEG coherence in individuals with autism. Biol Psyc, 62(3), 270‑273  Leno, Tomlinson et a., (2018): Resting‑state alpha power is selectively associated with autistic traits reflecting behavioral rigidity. Sci Rep, 8(1), 1‑7 |
| Auditory Oddball | Presents trains of standard  sounds followed by a deviant. | MMN/P3 ERP to deviants | Sensory processing  ERPs: Mismatch negativity | Näätänen, Sussman, Salisbur, & Shafer (2014): Mismatch negativity as an index of cognitive dysfunction. Brain Topog, 27(4), 451‑466  Slugocki & Trainor, (2014): Cortical indices of sound localization mature monotonically in early infancy. Eur J of Neurosci, 40(11), 3608‑3619 |
| Face ERP | Presents upright and inverted faces, objects and houses | P1/N170 ERP | Social processing  ERPs: Latency of face processing; face inversion effect (configural processing) | Eimer (2011): The face‑sensitive N170 component of the event‑related brain potential. The Oxford handbook of face perception, 28, 329‑44  Jones, Dawson & Webb (2018): Sensory hypersensitivity predicts enhanced attention capture by faces in the early development of ASD. Dev Cog Neurosci, 29, 11‑20 |

# Digital Biomarkers

Digital biomarker (dBM) technology allows for the remote measurement of the signs and symptoms of ASD, which can potentially reduce the burden of site visits and allow frequent/daily tracking in an ecologically valid environment. At screening, the caregiver will be provided with a smartphone app that will include a collection of digital biomarker assessments, including daily surveys and active tests, for the caregiver and participant to complete at home. In addition, the participant will be provided with passive monitoring technology that will measure behavioral and physiological signs of ASD symptoms.

*Additional Table 2: Schedule of events for the digital biomarkers*

| Visit |  | Titration Period | | | Treatment Period | | | |
| --- | --- | --- | --- | --- | --- | --- | --- | --- |
|  | screening | 1 | 2 | 3 | 4 | 5 | 6 | 7/ET^a^ |
| Informed consent | X |  |  |  |  |  |  |  |
| Identification of targeted behaviours for app | X |  |  |  |  |  |  |  |
| Hand over devices and training | X |  |  |  |  |  |  |  |
| Completion of In-Clinic Tasks^b^ | X | X | X | X | X | X | X | X |
| Completion of daily home tasks: |  |  |  |  |  |  |  |  |
| 1. Smartphone data collected through user interaction (Active Tasks)^c^ |  |  |  |  |  |  |  |  |
| 1. Data collected without user interaction (Passive Monitoring) |  |  |  |  |  |  |  |  |
| 1. Study Participant and Caregiver/Support Person Reported Outcomes, collected on smartphone (Surveys) |  |  |  |  |  |  |  |  |
| Review of compliance ^d^ |  |  |  |  |  |  |  |  |
| Return of digital biomarker devices and satisfaction survey^e^ |  |  |  |  |  |  |  | X |

ET=early termination or withdrawal visit; ^a^ To be conducted if a subject discontinues from the study or study drug treatment prematurely; ^b^ Due to the current pandemic (i.e., COVID-19), should visits not be able to take place in person, in-clinic tasks can be completed remotely on the day of the scheduled visit. In this case, the research team should contact the participant and caregiver by phone to give instructions and to ensure all the procedures are followed; ^c^ One Active Task should be completed each day by the study participant. In addition, the caregiver/support person should record a conversation with the study participant at least once per week and complete a numerical response scale to evaluate conversation quality. Active Tasks can be performed at days and times which the study participant and/or caregiver/support person deem convenient. Study participants are strongly encouraged to adhere to this schedule and collect as much data as they are able to. If participants find the schedule too burdensome, they may choose to reduce the assessment frequency without violating the protocol. ^d^ Participants will be encouraged to use the digital biomarker devices between study site visits; ^e^ Final satisfaction surveys on the participant’s experience about collection of digital biomarker will be completed by participants and by caregivers.
