# Supplementary material for: A Phase II Randomized, Double-Blind, Placebo-Controlled Study of the Efficacy, Safety, and Tolerability of Arbaclofen Administered for the Treatment of Social Function in Children and Adolescents With Autism Spectrum Disorders: Study Protocol for AIMS-2-TRIALS-CT1
Source: Front Psychiatry. 2021 Aug 24;12:701729. doi: 10.3389/fpsyt.2021.701729 (PMC8421761; doi:10.3389/fpsyt.2021.701729)
Supplement: Supplementary file 2 [file Data_Sheet_2.docx]

## Additional material 2

## Trial status

Currently under protocol v.7.2, dated 20.11.2020. Recruitment began on the 10.09.2019 and will be completed by mid‑2021 though the timeline might depend on the state of the Covid‑19 pandemic. At the time of submission of this manuscript, participants were still being recruited to the trial.

**Protocol amendments**

Protocols modifications have been undertaken in response to the needs of the study and/or response to the regulatory agencies in all three involved countries. To date, France has not submitted any amendment from their original submission. Spain has submitted 6 amendments and UK 8. Part of these amendments were improvements in the protocol before the recruitment started but also to adjust the protocol to the current pandemic situation.

**Ancillary and post-trial care**

There is no ancillary or post-trial care, but a 500,000 euros indemnity policy per patient is in place to cover any possible harm caused by the study.

**Confidentiality**

Privacy laws and regulations will be adhered to during all procedures related to this study. The collection and processing of participants’ personal information will be limited to what is necessary to insure the study’s scientific practicability. The information about potential candidates is shared between the clinician in charge and the responsible for this task in the research team and only after the patient/legal tutor verbally agrees to the clinician to inform the research team about the patient personal information for the purpose of being fully informed about the study. Information collected about participants during this clinical investigation will be treated confidentially. The local investigator or her/his co-workers will collect data and transfer it without recording the patient’s name or date of birth coded with a patient identification number. A patient identification code list linking the individual patients to the identification numbers will be kept at the site; access is restricted to authorized study team members. Pseudonymous (coded) data will be provided to Sponsor for scientific analysis or made available, if necessary, to the responsible supervisory authority (in case it audits/inspections the course of the study). Participants’ names will not be mentioned in any publication of study results. Persons monitoring the data will have access to all information needed to ensure the validity of the study data, and are required to keep information confidential and to respect data privacy.

**Dissemination policy**

The policy and plan for data analysis and publications for the current study is derived from policies and practices that were utilized in other multicenter academic studies and is in accordance with the principles and standards of scientific research and scholarship within the fields of biomedical research and scientific journalism. The goals of this policy are: a) to provide for the timely, scholarly and comprehensive reporting of the data in the scientific literature; b) to provide for the assignment of authorship and data analytic opportunities to study investigators in a manner that is equitable and supports career development and c) to ensure that the analysis and reporting data are consistent with regulatory agency requirements.

The data analysis and publication strategy will be determined by the Study Management Group. All study publications must be submitted for review to the Study Management Group. No treatment group information will be made available until after study completion. There is a specific Task (Task 4 in Work Package 1) in the project dealing with Communication/dissemination/exploitation strategy and awareness, with the involvement of all stakeholders including autistic participants.
