# Supplementary material for: A Phase II Randomized, Double-Blind, Placebo-Controlled Study of the Efficacy, Safety, and Tolerability of Arbaclofen Administered for the Treatment of Social Function in Children and Adolescents With Autism Spectrum Disorders: Study Protocol for AIMS-2-TRIALS-CT1
Source: Front Psychiatry. 2021 Aug 24;12:701729. doi: 10.3389/fpsyt.2021.701729 (PMC8421761; doi:10.3389/fpsyt.2021.701729)
Supplement: Supplementary file 4 [file Data_Sheet_4.docx]

# AIMS2-CT-01- Parent/Guardian Information Letter and Informed Consent

***Participants aged 5-17***

**EudraCT number generated is 2018-000942-21**

**A Randomized, Double-Blind, Placebo-Controlled Study of the Efficacy, Safety, and Tolerability of Arbaclofen Administered for the Treatment of Social Function in Children and Adolescents with Autism Spectrum Disorders.**

## Does treatment with arbaclofen help improving social function in Autism Spectrum Disorders?

You and your son/daughter are invited to take part in a research study. Before you decide whether or not to participate in this study, it is important to understand why this study is done. Also, it is important that you know the purpose of the study, the procedures, and possible risks and benefits of participation in this study. This is why we ask you to carefully read this information letter. Please do not hesitate to ask questions when the information is not clear. Take all the time you need to decide whether or not you wish to take part. It is up to you to decide whether or not to take part. If you do decide to take part you will be given this information letter to keep and will be asked to sign a consent form. If you decide to take part you and your son/daughter are still free to withdraw at any time, without giving a reason. Your decision will not affect the standard of care you receive from any medical services at any time.

### Why have we been invited to take part?

You have been invited to take part because your son/daughter is experiencing symptoms of an Autism Spectrum Disorder (ASD). Specifically, difficulties with relating to people and some behaviour difficulties. Children and adolescents from different European countries are invited by a researcher to take part in this study. In total, 130 children and adolescents with an ASD will take part in this project. You and your son/daughter are invited to take part in the AIMS-2-CT-01 study at [YOUR INSTITUTE].

### What is the purpose of this study?

Individuals with ASD have difficulties in social function. This includes understanding the nonverbal communication of other people, including gestures, facial expressions and non‑explicit intentions, and establishing good relationships with peers. The aim of the study is to determine whether arbaclofen helps in the treatment of these difficulties.

In this study, information is collected in several ways. Any symptoms your son/daughter may have are measured using interviews and questionnaires. Some of the interviews and questionnaires are administered to your son/daughter and some to yourself. We also take blood samples to look for some changes in chemical substances associated with social function. We also take registers of your son/daughter’s brain function with an electroencephalogram (EEG). The blood samples are used for safety issues and EEG will provide valuable information to enable us to understand the mechanism of action of arbaclofen. There is also an optional digital biomarker part of the study, for which we will provide your son/daughter with a study smartphone, a wrist-worn wearable device and several Bluetooth transmitters (Social Beacons). We hope that the study smartphone and wearable will help us measure important aspects of autistic behaviour at home, including repetitive behaviours (such as spending long times in a specific room doing the same activity over and over and again), cognitive abilities (as measured by some of the games in the mobile phone) and social communication skills (for example, a conversation between your son/daughter and yourselves). A few Social Beacons would be given to household members and placed around the home, allowing us to measure time spent with others and in social rooms (such as the living or the dining rooms, for example).

### Which treatment will be investigated?

Currently there is no medication approved for the core symptoms of ASD (difficulties in social function and specific autism behaviours). Arbaclofen is not an approved medication either. It has not been approved by any authority because there are not enough studies to support its approval. In this study we want to determine whether arbaclofen helps in the treatment of social function in ASD. We do this by treating people who suffer from this problem with arbaclofen or placebo daily. Half of the participants will receive tablets with arbaclofen. The daily dose will be a maximum of three tablets of 15mg if your son/daughter is younger than 12, and 20mg 3 times a day if your son/daughter is 12 years old or older. Arbaclofen is a product designed to modulate the equilibrium between two important substances of the brain, glutamate (excitatory) and GABA (inhibitory), by binding to one of the natural receptors of the GABA. Disruptions of the appropriate glutamate/GABA balance have been associated with autism, epilepsy and other neurodevelopmental disorders. Some research suggests beneficial effects of arbaclofen in children and adolescents with neurodevelopmental disorders including ASD. Half of the participants will receive a placebo. A placebo is a pill that does not contain an active substance meant to affect health. Whether your son/daughter receives arbaclofen or placebo is determined by chance, like rolling a dice. Neither you nor the research team will know which treatment your son/daughter receives until the end of the study. In the event of a serious side effect, we will open the blind and will know if your son/daughter was taking medication or placebo. The treatment with arbaclofen or placebo lasts 16 weeks.

### What will happen to my son/daughter if we take part?

The whole study takes 20 weeks and consists of 10 visits to the hospital. At the first visit (visit 0, screening) the researcher will explain the study to you and your son/daughter and ask you to sign the consent form at the end of this letter, for taking part in the study. Your son/daughter will be asked to assent either verbally or by signing an assent form. After this is done, you are asked some general questions such as your son/daughter’s age and medical history, and questions about his/her general health and any psychological problems that he/she may have. Interviews are conducted with you and with your son/daughter, some with both of you and some on a separate basis. Your son/daughter will be asked to perform some tasks. Some medical checks are performed, like measuring heart rate and blood pressure. A urine sample is taken and your son/daughter will have some blood samples taken. The second visit (visit 1, baseline) takes place within two weeks after visit 0. You are asked to complete some questionnaires. Your son/daughter will then have an EEG conducted. This will take about 4-5hrs. At the end of visit 1 your son/daughter receives the study medication. Between hospital visits, you will receive a telephone call to check on any incident that may have occurred.

We will ask you both to come back after 2 weeks (visit 2), 1 month (visit 3), 1½ months (visit 4), 2 months (visit 5), 3 months (visit 6), 4 months (visit 7) and 4½ months (visit 8, end‑visit). All visits involve interviews, questionnaires and a short medical check on your son/daughter. At visit 7 we will ask your son/daughter to have a second blood extraction, urine sample, and EEG. The screening visit will take around 3 hours, visit 1 and visit 7 may take around 4 or 5 hours and the rest of the visits may take around 1 or 2 hours. Two weeks after visit 8 we would like to see you to check that everything is ok after stopping having the medication. This visit will last 30mins.

During some of your visits, we would like to video- or audio-record part of the assessments and interviews. We record these assessments for quality control and a second rater (who does not know your son/daughter) to code some of the data. They will never be shared with third parties without your explicit permission. We will not audio- or video-record your son/daughter’s assessment unless you give explicit consent for us to do so.

If your son/daughter participates in the optional part of the study, he/she will wear a special wrist device that records his/her body movement, heart-rate, proximity to Bluetooth transmitters and location (GPS) during each day throughout the course of the study. The GPS signal is dereferenced, i.e. we record the distance and direction that the wearer travels, but never their actual location. The smartphone records the noise volume of the environment around the phone and time spent speaking, but never your son/daughter’s words or what you or other people around you say. In addition, your son/daughter will be asked to complete a couple of questions and simple tasks on the study smartphone on a daily basis. These tasks include a conversation with you that is recorded using the phone. The research team will help you to select the tasks based on your son/daughter’s ability level (for example, his/her age) to avoid tasks that are intended for older participants; it is possible that your son/daughter will complete no tasks or questions at all. The tasks take approximately 5 minutes per day. You or your son/daughter will be asked to help ensure that the collected data are transferred regularly by connecting the study phone to your home WiFi when you first return home and leaving it connected throughout the study. You are also asked to fill out a survey related to some of your son/daughter’s recent behaviors on visits1 and 7. This questionnaire will take 10mins.

Finally, we would like to contact your son/daughter’s teacher to complete a short questionnaire for us at 2 different times. We will also ask you if this is ok with you.


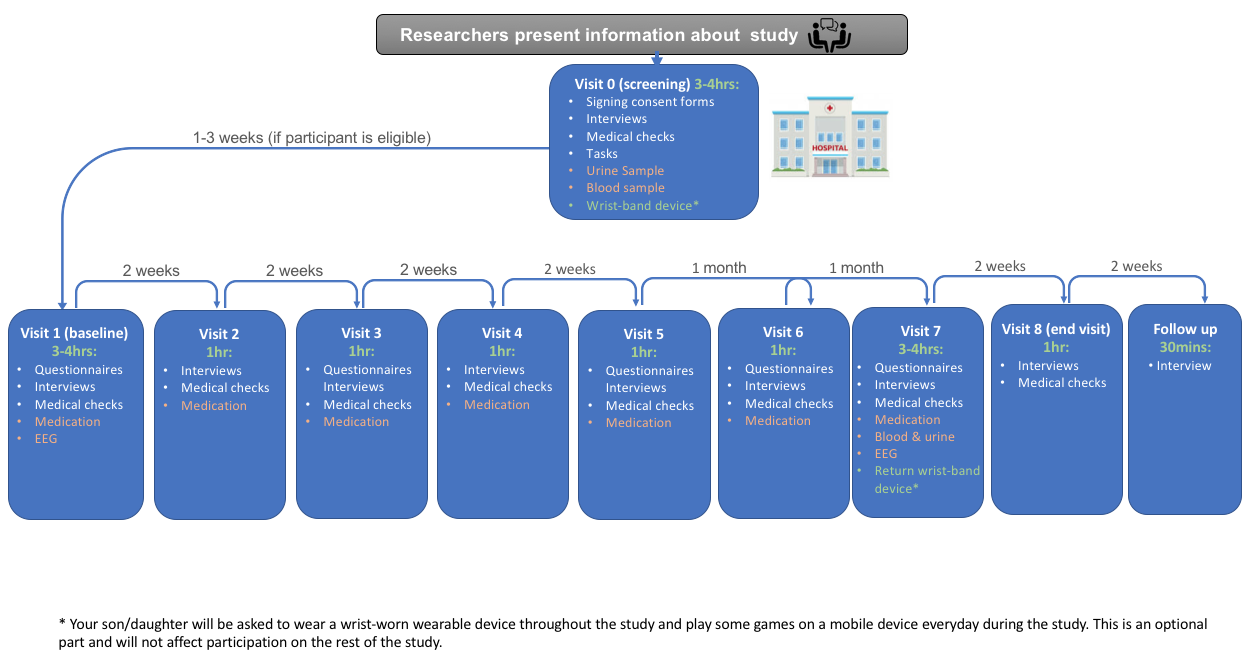


### What do we have to do?

- Intake of study medication

At the start of the study your son/daughter will receive sufficient tablets until the next visit. We will explain you both in detail how many tablets you will have to take every day. If your son/daughter is under 12 years of age he/she will take 1 tablet per day in the first week, 2 tablets per day in the two following weeks, and 3 tablets per day from the fourth week on. If your son/daughter is 12 years old or older, he/she will take 2 tablets per day the first two weeks, and 3 tablets per day from the third week on. This intake regime may vary slightly, depending on how he/she tolerates the tablets. Please make sure your son/daughter takes the tablets during a meal. ***Tablets dissolve on the tongue, do not break, crush or chew them. He/She does not have to break or bite it****.* It is important that your son/daughter does this every day. This is why the researcher will discuss this at every visit. In every visit your son/daughter will get sufficient medication to take until the following visit. The tablets should stay in the blisters until use, so the use of pill boxes is not allowed during the trial.

- Use of other medication during the study

There are some medications that are not compatible with the medication of this clinical trial because they can influence the effect of the study medication. This is why the researcher will ask you at every visit about your son/daughter’s use of other medication, for instance medication that is prescribed by your general practitioner or any medication you have bought at the drug store. It is important that you tell the researcher what medication your son/daughter is using. Medications that must not be used while your son/daughter is in the clinical trial include racemic baclofen, vigabatrin, tiagabine, or riluzole or other GABA-related medications such as gabapentin or pregabalin. Benzodiazepines (or derivatives) can only be used at night and on an occasional basis. If your son/daughter is on antiepileptic medication, this should be stable for the duration of the trial. Please, show this list of incompatible medications to any other doctor that you may visit during the course of this trial.

- Use of alcohol and drugs during the study

Alcohol, cannabis and other drugs have an effect on your son/daughter’s mental health and can influence the effect of arbaclofen. Therefore it is important to tell the researcher about the use of one of these substances. The researcher will ask your son/daughter about this regularly.

- Benzodiazepine use before EEG

The use of benzodiazepines (or derivatives) may interfere with the EEG register. Please do not use benzodiazepines the night before your son/daughter will have the EEG (visits 1 and 7).

- Pregnancy during the trial

Arbaclofen has not been tested in pregnant women. To avoid exposure of an unborn baby to the medication, you must use birth control (contraception) if you are sexually active.

For female participants who are able to become pregnant:

If your daughter is pregnant, becomes pregnant, or is currently breastfeeding, she cannot take part in this study. Female participants must agree to use a reliable form of contraception (complete abstinence or a combination of two effective forms of contraception, such as for example, condoms plus hormonal treatment) during the study and for five months after the last dose of study medication. The study doctor or nurse will discuss contraceptive methods in more detail with you. For safety reasons, we will ask female participants of childbearing potential to have a pregnancy test at screening, visit 3, 5, 6 and 7. If you suspect your daughter is pregnant, if she becomes pregnant during the study, or within five months of your last study medication treatment, you must inform us immediately. In that case, we will perform an extra pregnancy test to confirm. If the result is positive, your daughter will have to stop having the medication.

For male participants:

Your son should use an acceptable method of birth control or practice complete abstinence with any female sexual partners who could become pregnant. It is important that he does this during the study and for an additional seven months after treatment has finished. The study doctor or nurse will discuss contraceptive methods in more detail with you. If your son has a female sexual partner and gets pregnant, we would like to ask her to follow her pregnancy **[delete if your local regulations do not require so].**

- Special measures following COVID-19

Due to the current world-wide situation, and in order to maintain the safety of both families and research staff, some assessments will be able to be conducted remotely, if deemed safer, via end-to-end encrypted videoconference facilities that your clinician will arrange with you.

### What side effects can we expect?

There is not much information about the side effects of arbaclofen. In the only controlled study to date, conducted with 150 participants, the side effects were mild and not dangerous. More frequently than placebo arbaclofen may cause some affect lability or somnolence. Other side effects have not been observed more frequently than with placebo. You can check the full list of side effects observed in that study in the APPENDIX 1 of this consent form. Please contact the study team or your doctor when you experience significant side effects. The study will be discontinued if the side effects you report are considered severe.

### What are the possible benefits of taking part?

Previous studies found that the use of arbaclofen may improve social function in adolescents with ASD. In addition, you will contribute to the development of medical knowledge from which your son/daughter and other people with these disorders could benefit in the future.

### What are the possible risks of taking part?

- Arbaclofen treatment

As described before, treatment with arbaclofen could cause harmless side effects.

- EEG

The EEG procedure is painless and safe, and there are no known health risks. It involves putting a cap on your head in order to attach some cables to it; this may be felt as slightly uncomfortable to some participants. A member of the study team will be in constant contact with your son/daughter during the EEG procedure. If he/she feels uncomfortable in any way the EEG can be stopped. There is no radiation involved.

- Blood sample

The risks of the blood taking in this study are the same as those of ordinary blood draws. There could be small mild discomfort, and occasionally a small bruise or minor swelling at the site of the injection. These symptoms can be soothed with an ice pack. Light-headedness or dizziness may happen occasionally. This risk will be minimized by ensuring that the blood sampling is performed by trained researchers. Only a small amount of blood is taken per visit (15mls maximum).

- Study smartphone, Bluetooth transmitters and wrist-worn device

The assessments at home using the study smartphone and other sensors (Bluetooth transmitters and wrist-worn device) are not expected to cause harm or major inconvenience to the participants.

### What happens if we do not want to participate?

It is up to you and your son/daughter to decide whether or not to take part in this study. Participation is voluntary. When you both decide to participate, you are free to change your mind and to stop with the study at any time, also during the study. When you decide to stop, nothing else is expected from you. You do not have to sign for this decision. Also, you do not have to explain why you want to stop. You and your son/daughter will receive the treatment you would normally receive.

### What happens when the study is ended?

Your participation in the study stops when you and your son/daughter have completed all the visits or when you choose to stop. Also, the ethics committee or the sponsor can stop the study, or the researcher can decide to stop your participation. This can be decided without your consent. This happens only because of medical or scientific reasons. The safety and wellbeing of your child is continuously monitored; if his or her wellbeing is in danger, we will discontinue the study immediately. The study is concluded once all the participants have completed the study.

### Are we insured when we take part in this study?

There is an insurance for every study participant. The insurance covers possible damage as a result of your participation in this study. This insurance complies with the Spanish legislation *Real Decreto 1090/2015*) [ADAPT TO YOUR SITE] and concerns harm that comes to you and your son/daughter in relation with your participation in the study, provided that it is not a consequence of the disorder under study. If you need more information regarding safety you can consult the principal researcher about it.

### Will we be informed when relevant information becomes available during the study?

We aim to perform the study according to plan. But the situation can change. For example, because of new information about the study medication. When this happens, we will immediately discuss this with you and your son/daughter. At that moment you both can decide to stop or to continue your participation.

### What happens if there are any incidental findings?

It is possible that during the study new knowledge is obtained that is important for your son/daughter’s health. This can happen for example during a medical check. In this case a doctor will explain to you and your son/daughter what it is, and will provide you with advice on what should be done. If necessary, your son/daughter will be referred to an appropriate physician. The type of EEG assessment that we are doing as part of the study does not allow us to draw any clinical conclusions, hence we will not be able to provide you with any feedback on or report incidental findings related to the EEG recordings. [PLEASE ADJUST THIS IN LINE WITH LOCAL REQUIREMENTS, IF APPLICABLE]

### What happens with our information?

All information we collect from your son/daughter in this study is strictly confidential. His/her personal information will be de-personalised. This means that his/her personal information will be replaced by a number. The data and any bodily material collected by the Sponsor of the study will only contain this number. Data can only be traced back to you with an encryption key linking the number to your personal information. This encryption key remains safely stored at the local institute and can only be accessed by a small number of people working on this study. Thus, nobody who looks at the results will know your son/daughter’s name, or any other personal details that could be used to identify him/her. The medical file will not leave the hospital or institution that your son/daughter visits. The file can be seen and examined by employees who are involved in this study. These persons check whether the study is carried out properly and reliably. Monitors from the sponsor, persons auditing the study, members of the Ethical Review Board or other regulatory authorities are allowed to examine the files. If you sign the informed consent form, you give permission to grant these individuals access to your medical data relevant for the study. They will keep your data confidential. The results are stored in a highly secured environment and privacy laws will be adhered to. If you decide to withdraw from the study, you have the right to claim that any information we have collected from your son/daughter will be destroyed. We are required to store his/her study information for 25 years.

### What happens with my son/daughter’s blood samples?

All blood samples are stored pseudo-anonymized, with only the number as explained above. We would like to keep some of your son/daughter’s blood samples. We may be able to use them for additional research in the future. This will concern research in the context of ASD. You and your son/daughter will be presented with another Consent Form in case we want to use his/her samples for other studies beyond this clinical trial or for any genetic study. You can always withdraw this consent; in this case, his/her blood samples will be destroyed. If you do not give consent for this, your son/daughter can still participate in the current study.

As part of collaborations in this study, your son/daughter’s data may be transferred for analysis to other research groups, possibly in other countries. Outside the EU, other rules may apply for the protection of personal data and this will always be oversaw by the principal investigator of the clinical trial and the appropriate Ethics Committee. Therefore, you and your son/daughter will be asked at the end of this consent form to give consent to transfer his/her data. Only coded data (i.e. your personal information is replaced by a number) and/or blood samples will be transferred [PLEASE ADJUST THIS IN LINE WITH LOCAL REQUIREMENTS, IF APPLICABLE – DELETE IF GENETIC CONSENT IS ASKED SEPARATELY]. If you do not give consent for this, your son/daughter cannot participate in the study.

### We would also like to ask your permission for sharing your don/daughter’s anonymized data with other research groups beyond this study. Sharing data beyond the AIMS trial is optional. We may be able to use your son/daughter’s data for additional research in the future. This will concern research in the context of ASD. If you do not give consent for this, your son/daughter can still participate in the study.

### What happens with my data provided via the optional study smartphone, Bluetooth transmitters or wrist-worn device?

For privacy reasons, no personally identifying information is stored on the devices or the secure servers where the data is stored. Audio is recorded during the speech task and the conversation recording task; it is stored unaltered. For the conversation recording task, it is possible to delete part or all of the recording if, for example, only background or ambient noise is recorded, or you choose to delete the recording for privacy reasons. You have the option to pause ambient noise recorded by the smartphone.

The data collected from you and your child are encrypted, meaning that data are converted to an unreadable format and can only be accessed by password that is only accessible by selected research members to match the data to the other measures collected during the study. The data are transmitted from the phone to a secure server by WIFI via a secure connection. As part of the collaboration with the company providing these devices (F. Hoffmann La-Roche, Ltd.), your data would be transferred to F. Hoffmann La-Roche, Ltd for processing, storage and analysis and to research members in the sponsoring team. For this purpose, the data may be stored in the US, in the European Union, the UK, or in Switzerland. This also applies to the data collected during the conversation task (if you agree to do it), which means that your voice may be heard by people outside the study/sponsor team. Outside the EU, different rules may apply for protection of data and this will always be overseen by the sponsor. Only encrypted password-protected data from these devices will be transferred.

You will be asked as an option at the end of this consent form to give consent to participate in this digital biomarkers part of the study and to transfer your data indicated above. Saying no to this part of the study will not affect your participation in the rest of the study. If you decide to participate, and similarly to other type of data collected for this study, data will be kept for 25 years.

### Will our GP be informed?

We will let your GP know that your son/daughter participates in the study, in writing. This is for your son/daughter’s own safety. You and your son/daughter have to give consent for this in the consent form. When you do not provide consent, you cannot participate in this study. [PLEASE ADJUST THIS IN LINE WITH LOCAL REQUIREMENTS, IF APPLICABLE]

### Will we be paid if I take part in this study?

We will financially compensate you for the time spent on this study. This is an amount of [please amend according to local procedures in participant reimbursement] if you complete all study visits and procedures. If you withdraw from the study earlier this amount will be adjusted to the extent to which you have participated in the study. Also travel costs will be reimbursed. [Please amend according to local procedures in participant reimbursement].

### Who has reviewed the study?

This study is reviewed and approved by the Ethical Review Board of [YOUR INSTITUTE]. This committee has reviewed the study for compliance with medical and ethical standards and for scientific value.

### Public information

The information that this study is taking place will be available in publicly available registries: clinicaltrials.org and the “Registro Español de Ensayos Clínicos, REEC”. [PLEASE ADJUST THIS IN LINE WITH LOCAL REQUIREMENTS, IF APPLICABLE].

### Who do we contact if we need further information?

For general information about your or your son/daughter’s rights when processing your personal data, you can consult the website of the [COUNTRY] Data Protection Authority (<https://www.gov.uk/data-protection>).

If you have any questions about your rights, please contact the person responsible for processing of your personal data. For this study that person is [ADD PERSON IN LINE WITH GDPR]. You can also contact the Sponsor, who is responsible for the data collection of the study as a whole. For this study that person is Dr Celso Arango ([carango@hggm.es](mailto:carango@hggm.es)). If you have questions or complaints about the processing of your personal data, you can contact the Data Protection Officer of the institute [ENTRE YOUR INSTITUTION NAME]. This person is [ENTER RESPONSIBLE PERSON´S NAME and details]. If you or your son/daughter have any questions about the research, your rights as a parent/participant, or would like to report any problem or injury arising from the research, please contact [Name researcher and telephone number]. You can also ask any questions to an independent physician, who is not part of the study, but has enough knowledge about the study to answer your questions [ENTER NAME AND CONTACT DETAILS]

**Appendix 1: Side effects table**

Treatment-Emergent Adverse Events^a^ (AEs) from Baseline to week 12 reported in the Seaside RCT ([Veenstra-VanderWeele et al., 2017](#_ENREF_65))

| Event | Placebo N = 74  *n* (%) | Arbaclofen N = 76  *n* (%) |
| --- | --- | --- |
| Any adverse event | 58 (78.4%) | 64 (84.2%) |
| Vomiting | 7 (9.5%) | 12 (15.8%) |
| Upper Respiratory infection | 10 (13.5%) | 10 (13.2%) |
| Affect lability | 1 (1.4%) | 8 (10.5%) |
| Headache | 4 (5.4%) | 8 (10.5%) |
| Irritability | 8 (10.8%) | 7 (9.2%) |
| Somnolence | 1 (1.4%) | 7 (9.2%) |
| Insomnia | 10 (13.5%) | 7 (9.2%) |
| Aggression | 5 (6.8%) | 6 (7.9%) |
| Diarrhoea | 6 (8.1%) | 6 (7.9%) |
| Decreased appetite | 2 (2.7%) | 5 (6.6%) |
| Hyperactivity | 6 (8.1%) | 5 (6.6%) |
| Anxiety | 5 (6.8%) | 4 (5.3%) |
| Sleep disorder | 3 (4.1%) | 4 (5.3%) |
| Weight decreased | 2 (2.7%) | 4 (5.3%) |
| Nasopharyngitis | 5 (6.8%) | 4 (5.3%) |
| Cough | 3 (4.1%) | 4 (5.3%) |
| Nasal congestion | 1 (1.4%) | 4 (5.3%) |
| Pyrexia | 6 (8.1%) | 3 (3.9%) |
| Agitation | 4 (5.4%) | 2 (2.6%) |
| Rhinorrhoea | 6 (8.1%) | 2 (2.6%) |
| Rash | 5 (6.8%) | 2 (2.6%) |

**AIMS2-CT-01- Informed Consent Form**

***Participants aged 5-17***

**A Randomized, Double-Blind, Placebo-Controlled Study of the Efficacy, Safety, and Tolerability of Arbaclofen Administered for the Treatment of Social Function in Children and Adolescents with Autism Spectrum Disorders.**

## Does treatment with arbaclofen help improving social function in Autism Spectrum Disorders?

By signing this document I declare the following:

1. **I have read the information letter and I have been given a copy.** I was given the opportunity to ask questions. My questions are answered to my satisfaction. I understand why the research is being done and the risks involved. I had enough time to decide for me and my son/daughter whether to take part.
2. **I understand that the participation is voluntary.** **I understand that we are free to withdraw consent at any time**, without giving any reason, without the medical care or legal rights being affected. When we withdraw consent, all collected information can be destroyed.
3. **I give permission to inform my son/daughter’s general practitioner about participation**. [PLEASE REMOVE THIS IF THIS IS NOT REQUIRED LOCALLY].
4. **I understand that relevant sections of my son/daughter’s medical records and data collected during the study may be looked at by individuals from the research team, from regulatory authorities or from the Sponsor of the study.** I understand that they can ask questions about my son/daughter’s medical well-being to health professionals involved in my care. I give permission for these individuals to have access to his/her records and to contact the relevant mental health professionals.
5. **I understand that the results from this study may be used commercially** and that we will not get money from this research.
6. **I give consent to store my son/daughter’s records for 25 years after the end of this study for the purposes of this research study.** I understand that such information will be treated as confidential and handled in accordance with applicable data protection legislations.
7. **I am satisfied that my son/daughter’s welfare and interests have been properly safeguarded** in asking to donate biological tissues for research in the above project.
8. **I agree to the audio- and video-recording of some assessments with me/my son/daughter** and that these recordings will be used for the purposes of data coding and quality monitoring of this research. I understand I will be consulted before this recording is shown to a wider audience.
9. I give consent to transfer my son/daughter’s data **and/or blood samples [DELETE IF GENETIC CONSENT IS ASKED SEPARATELY]** **as part of this study** to other research groups, possibly in other countries. I know that other privacy rules may apply outside the EU. Only coded data and/or blood samples will be transferred, without name and other personal information that can identify my son/daughter.
10. I agree to inform the study team if I suspect my daughter is pregnant. I understand that in that case, the study team can terminate her participation in the study if the pregnancy is confirmed.
11. I agree for my and my son/daughter to be interviewed via end-to-end encrypted videoconference facilities or telephone when this may be in the interest of everyone’s safety.

**OPTIONAL [KEEP OR DELETE ACCORDING TO LOCAL REQUIREMENTS]:**

Please initial the box if applicable:

1. **I agree that my son/daughter’s blood samples will be stored in a pseudo-anonymized fashion at the study centre for use in future projects [CHECK LOCAL REQUIREMENTS FOR SEPARATE CONSENT]**. I understand that some of these projects may be carried out by researchers other than those who ran the first project, including researchers working for other universities within or outside Spain. I understand that the use of samples by institutions other than those running the first project will be subject to ethical review and subject to my explicit consent at the time of such study **[Adapt to local requirements (delete this sentence if consent for genetics is included in the same consent form and not handled separately)].**
2. **I give permission for my son/daughter’s data to be shared in an anonymised fashion** **with the scientific community outside of the AIMS2TRIALS group of research institutes** **for future research related to Autism Spectrum Disorders beyond this trial**. I understand that some of these projects may be carried out by researchers other than those who ran the first project, including researchers working for other universities within or outside Spain.
3. **We can be contacted again in the future** should further information be required. I understand that any future help is voluntary.
4. We can be contacted again in the future to take part in other similar studies. I understand that my son/daughter and I can refuse to take part in follow up research without giving a reason and without medical treatment or legal rights being affected.
5. **I agree for my son/daughter to take part in the optional Digital Biomarkers element of this study.** I understand that coded data collected will be used as part of this study and may also be transferred to and used by other researchers working for other universities within or outside the EU as well as by the provider of the equipment (F. Hoffmann La-Roche, Ltd.).

**I agree to my and my son/daughter’s participation in this study.**

Name of participant (child/adolescent):……………………………………………………………

Date of birth: __ / __ / __

Name of parent / legal guardian:…………………………………………………………………..

Signature: Date: __ / __ / __

**FOR THE RESEARCH TEAM TO COMPLETE:**

I declare that I fully informed the above-mentioned persons about this study.

Name researcher: ………………………………………………………………………….

Signature: Date: __ / __ / __

---------------------------------------------------------------------------------------------------------------------------

The above-mentioned persons asked questions of a medical nature Yes

No

I declare that I fully informed the above-mentioned persons about any medical issues related to this study.

Name physician: ………………………………………………………………………….

Signature: Date: __ / __ / __

---------------------------------------------------------------------------------------------------------------------------
