# Supplementary material for: A Phase II Randomized, Double-Blind, Placebo-Controlled Study of the Efficacy, Safety, and Tolerability of Arbaclofen Administered for the Treatment of Social Function in Children and Adolescents With Autism Spectrum Disorders: Study Protocol for AIMS-2-TRIALS-CT1
Source: Front Psychiatry. 2021 Aug 24;12:701729. doi: 10.3389/fpsyt.2021.701729 (PMC8421761; doi:10.3389/fpsyt.2021.701729)
Supplement: Supplementary file 5 [file Data_Sheet_5.docx]

## Additional material 5

**SOP Training Requirements (version 3.1 dated 2020, Dec 23^rd^) for ADOS-2 raters**

“The clinician/researcher must be reliable for its administration and coding and maintain reliability of at least 80% agreement with a local consensus group. Each site should have an ADOS lead (could be external) and regular ADOS reliability meetings should be attended. The ADOS lead is a local ADOS-2 expert (with official training certification for research purposes). Other ADOS administrators should have followed a certified ADOS-2 research training or, alternatively, be supervised by a well-trained study team member, as confirmed with a signed, ADOS training certificate. The ADOS lead is responsible for the training and supervision of his/her team members and for the local consensus meetings they hold. Reliability of the team member administering the ADOS with the consensus scores derived from the supervision meetings should be recorded. Reliability above 80 % should be reached in at least 2 consecutive ADOS-2 administrations before a given team member is allowed to administer and score independently.

Any deviation from reliability should be studied at each site and, in agreement with the ADOS lead of that site, corrective measures be taken (e.g. practice more ADOS, etc.). The authorization to perform the ADOS, should be documented in one of two ways:

1) Confirmation of training (for research purposes) of unexperienced team members: own, local training certificate. If not available, complete the template ADOS certificate provided, signed off by your local ADOS lead.

2) Confirmation of training (for research purposes) of ADOS lead: a signed certificate from an official ADOS training.”
